# Contribution of Multiple Inter-Kingdom Horizontal Gene Transfers to Evolution and Adaptation of Amphibian-Killing Chytrid, *Batrachochytrium dendrobatidis*

**DOI:** 10.3389/fmicb.2016.01360

**Published:** 2016-08-31

**Authors:** Baofa Sun, Tong Li, Jinhua Xiao, Li Liu, Peng Zhang, Robert W. Murphy, Shunmin He, Dawei Huang

**Affiliations:** ^1^Key Laboratory of Zoological Systematics and Evolution, Institute of Zoology, Chinese Academy of SciencesBeijing, China; ^2^CAS Key Laboratory of Genomics and Precision Medicine, Beijing Institute of Genomics, Chinese Academy of SciencesBeijing, China; ^3^Key Laboratory of Crop Pests Control of Henan Province, Institute of Plant Protection, Henan Academy of Agricultural SciencesZhengzhou, China; ^4^Network & Information Center, Institute of Microbiology, Chinese Academy of SciencesBeijing, China; ^5^Department of Natural History, Royal Ontario MuseumToronto, ON, Canada; ^6^Shandong Provincial Key Laboratory for Biology of Vegetable Diseases and Insect Pests, College of Plant Protection, Shandong Agricultural UniversityTai'an, China

**Keywords:** horizontal gene transfer, fungal pathogen, amphibian, evolutionary analysis, purifying selection

## Abstract

Amphibian populations are experiencing catastrophic declines driven by the fungal pathogen *Batrachochytrium dendrobatidis* (*Bd*). Although horizontal gene transfer (HGT) facilitates the evolution and adaptation in many fungi by conferring novel function genes to the recipient fungi, inter-kingdom HGT in *Bd* remains largely unexplored. In this study, our investigation detects 19 bacterial genes transferred to *Bd*, including metallo-beta-lactamase and arsenate reductase that play important roles in the resistance to antibiotics and arsenates. Moreover, three probable HGT gene families in *Bd* are from plants and one gene family coding the ankyrin repeat-containing protein appears to come from oomycetes. The observed multi-copy gene families associated with HGT are probably due to the independent transfer events or gene duplications. Five HGT genes with extracellular locations may relate to infection, and some other genes may participate in a variety of metabolic pathways, and in doing so add important metabolic traits to the recipient. The evolutionary analysis indicates that all the transferred genes evolved under purifying selection, suggesting that their functions in *Bd* are similar to those of the donors. Collectively, our results indicate that HGT from diverse donors may be an important evolutionary driver of *Bd*, and improve its adaptations for infecting and colonizing host amphibians.

## Introduction

Globally, amphibian populations are facing massive declines due to many factors. One insidious driver of the catastrophic die-offs is the emerging global pathogen *Batrachochytrium dendrobatidis* (*Bd*), which is a chytrid fungus (Longcore et al., 1999; Hof et al., 2011). This fungus, which causes chytridiomycosis, occurs in hundreds of amphibian species (Berger et al., 1998), (Skerratt et al., 2007; Wake and Vredenburg, 2008). Most studies on *Bd* have focused mainly on its ecology and population genetics. The molecular mechanism of its infection and lethality remains largely unexplored (Morehouse et al., 2003; Morgan et al., 2007). The evolutionary position of the poorly characterized Chytridiomycota presents a major challenge to understanding this fungal species. Chytrids are basal fungi separated by a vast evolutionary distance from any well-characterized relatives (James et al., 2006; Rosenblum et al., 2008). Fortunately, the Joint Genome Institute and Broad Institute sequenced complete genomes of the *Bd* strains JAM81 and JEL423, respectively. These genomes data facilitate genomic investigations of molecular mechanisms of their infection lifestyle.

Horizontal gene transfer (HGT) involves the transmission of genetic material across species boundaries. It is an important evolutionary driver of the genomes of many organisms because one organism can acquire novel functional genes rapidly from another organism. Such newly acquired genes accelerate the adaptation and evolution of the recipients (Mitreva et al., 2009; Richards et al., 2011b). Horizontal gene transfer has been extensively studied and its significance in prokaryotic evolution is well known (Doolittle, 2005; Boucher et al., 2007; Dagan et al., 2008; Dorman and Kane, 2009). HGT also contributes significantly to evolution of fungi and other eukaryotes, although knowledge about HGT in eukaryotes is limited (Keeling and Palmer, 2008). A variety of cases are known among fungi (Richards et al., 2011a), including single-gene (Strope et al., 2011), gene clusters (Khaldi et al., 2008; Slot and Rokas, 2010, 2011; Campbell et al., 2012) and entire chromosomal transfers (Rosewich and Kistler, 2000; Ma et al., 2010; van der Does and Rep, 2012). Fungi also can acquire functional genes from organisms in other kingdoms such as bacteria, viruses, plants, and animals (Rosewich and Kistler, 2000; Marcet-Houben and Gabaldón, 2010; Fitzpatrick, 2011; Richards et al., 2011a). Horizontal gene transfer can yield instant advantages to fungal metabolism, propagation and pathogenicity and in doing so bestow significant selective advantages (Marcet-Houben and Gabaldón, 2010; Fitzpatrick, 2011; Richards et al., 2011a). Among the inter-kingdom HGT cases, fungi most frequently acquire novel genes from bacteria. Though exceedingly rare, fungi also can acquire genes from plants and animals (Richards et al., 2009; Selman et al., 2011; Pombert et al., 2012; Zhao et al., 2014).

A recent HGT study on *Bd* revealed that two large families of known virulence-effector genes, crinkler (CRN) proteins and serine peptidases, were acquired by *Bd* from oomycete pathogens and bacteria, respectively (Sun et al., 2011). These two gene families have duplicated and evolved under strong positive selection, which may relate to the virulence of *Bd* to its amphibian hosts. It is probable that *Bd* acquired other important functional genes via HGT, facilitating its adaptation of pathogenic lifestyle. To address this possibility, we focused on inter-kingdom HGT by analyzing protein sets of the two *Bd* strains and exploring gene transfer from suites of non-fungi species ranging from viruses, bacteria, protists, plants and animals. We use comprehensive homology searching and phylogenetic analyses to detect all probable HGT candidates and then analyze their functional and evolutionary contributions to *Bd*. We discovered that many bacteria-derived genes exist in *Bd* in addition to serine peptidases, three transferred genes appear to have botanical origins and the gene family coding the ankyrin repeat-containing protein may originate from oomycetes. No credible evidence indicates HGT from host amphibians. Some functional genes involve multiple transfers yet others duplicated subsequent to their HGT. Functional analyses indicate horizontally transferred genes appear to play important physiological roles in *Bd*. Overall, HGT from diverse donors may be an important evolutionary driver of *Bd*.

## Materials and methods

### Data sources

We retrieved 8700 and 8818 predicted proteins of *Bd* JAM81 and JEL423 from NCBI (http://www.ncbi.nlm.nih.gov/protein) and the Broad Institute (http://www.broadinstitute.org/annotation/genome/batrachochytrium_dendrobatidis/MultiDownloads.html), respectively. Further, we downloaded protein sequences in the RefSeq of NCBI (ftp://ftp.ncbi.nlm.nih.gov/refseq/release/) for a wide diversity of bacteria, fungi, protozoans, viruses, plants, and animals. We constructed two local proteome databases for the RefSeq sequences. One database contained all proteins of fungi with completed genomes in RefSeq and the other one was comprised of all the proteomes excluding fungi. The whole-genome expression assays data of *Bd* JAM81 with GEO accession number GSE37135 (http://www.ncbi.nlm.nih.gov/geo/query/acc.cgi?acc=GSE37135) were downloaded to quantify expression levels of horizontally transferred genes (Rosenblum et al., 2012).

### Screening for HGT candidates

**Figure 1:**
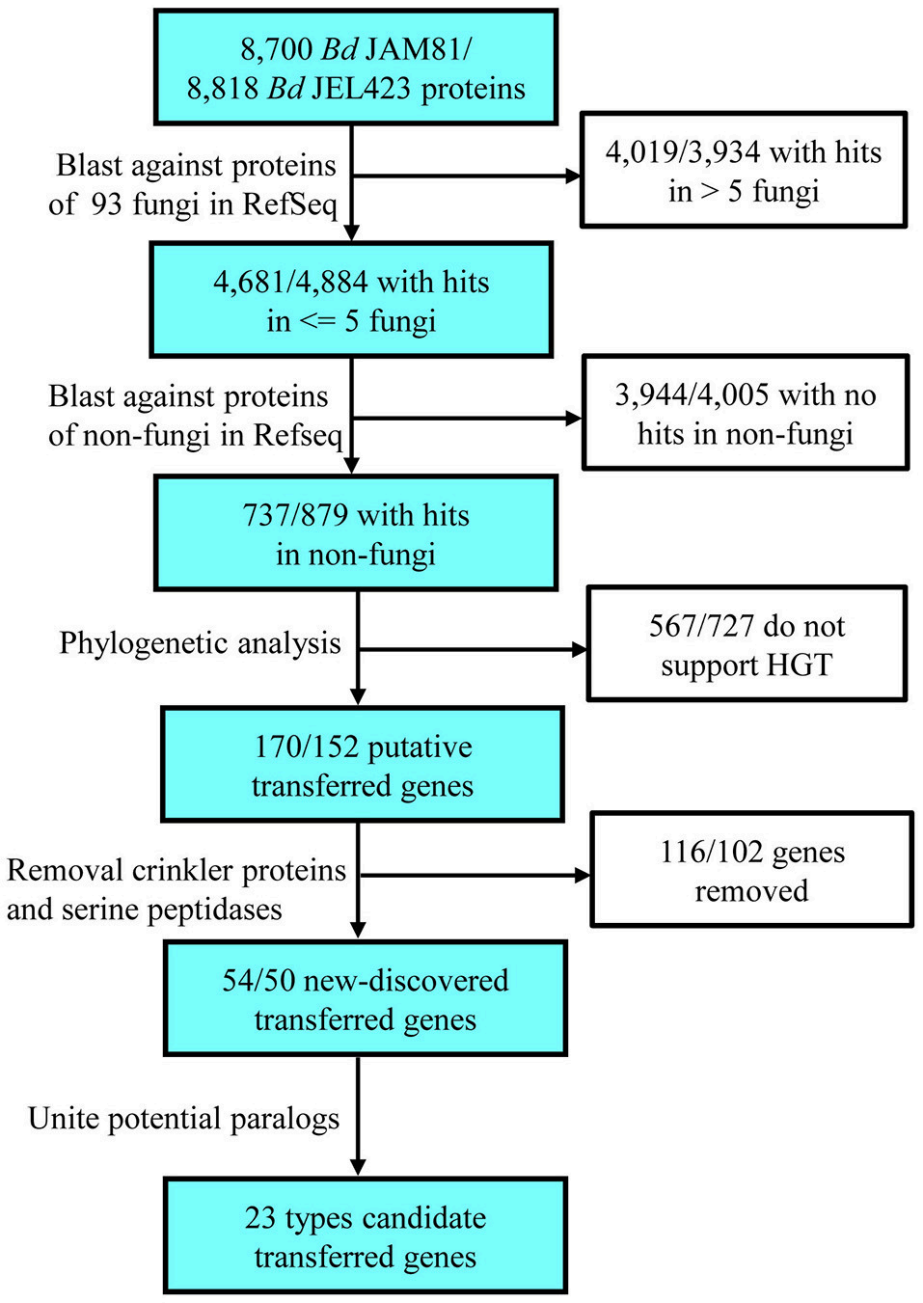
**Flowchart used to search for horizontally transferred genes in *Bd* and the results of each step**. In total, 23 types of transferred genes were detected in *Bd*.

A multi-step bioinformatic pipeline was used to identify candidate HGTs (Figure 1 and Table S1). Blast (Altschul et al., 1997) was employed to screen proteins present in only a few fungal species but widespread in other groups. First, each protein sequence occurring in both *Bd* strains was compared with fungal sequences in the local database using BLASTP. Blast results were filtered based on an e-value threshold of 1e^−10^ and a continuous overlap threshold of 33% with the query protein. Proteins with homologs in five or fewer fungi were selected as possible HGTs involving *Bd* only as well as ancient HGTs (Marcet-Houben and Gabaldón, 2010). These candidates were then compared to the non-fungi database via BLASTP with the same e-value threshold. Next, we constructed the phylogeny of each protein with homologs in more than 20 non-fungal species to validate the HGT (see below).

To exclude possible DNA contaminates, the two *Bd* strains were assumed to be independently evolving entities and, therefore, unlikely to host identical contaminates. Accordingly, we retained candidate genes present in both strains. Gene phylogenies were generated to detect the origins of candidates. Vertical inheritance was assumed to be generally congruent with the species' phylogeny such that a putative HGT gene was physically linked to native genes on the genome of *Bd* (Sun et al., 2011). Further, because bacteria lack introns, we retrieved the number of exons in potentially horizontally transferred genes from gff files of the genome sequences.

### Phylogenetic analyses

Homologs of horizontally transferred genes were searched in non-redundant databases by BLASTP (*e* = 1e^−10^). Next, homologous protein sets were submitted to multiple alignments using ClustalW2 (Thompson et al., 1994). Subsequently, the alignments were inspected visually and refined manually. Phylogenetic analyses were performed with Bayesian inference (BI) and maximum likelihood (ML). We also constructed distance-based neighbor-joining (NJ) trees.

Bayesian trees were generated by MrBayes 3.1.2 (Ronquist and Huelsenbeck, 2003). Each candidate HGT was submitted to two independent analyses using four MCMC chains based on models of amino acid substitution estimated by Prottest 3.0 (Abascal et al., 2005). We ran analyses for one million generations with a sampling frequency of 100 generations. Analyses were stopped when the average deviation of split frequencies was less than 0.01. The initial 25% of the total trees was discarded as burn-in. Compatible groups were shown in the majority rule consensus tree. Maximum likelihood trees were constructed using Phyml 3.1 (Guindon et al., 2010) with the best-fit evolutionary model suggested by Prottest 3.0 (Abascal et al., 2005). Branch support values were gained by performing 1000 bootstraps. NJ trees were constructed using Neighbor in MEGA6 (Tamura et al., 2013). Bootstrap values were obtained by generating 1000 pseudoreplicates. HGT was inferred when the topology within a well-supported clade contained non-sister species. Direction of transfer was inferred from the distribution of the horizontally transferred gene and its placement on the trees.

### Evolutionary mapping of HGTs

Two scenarios could explain the presence of homologs for some horizontally transferred genes: multiple transfer events from different donors and gene duplication subsequent to a transfer. Homology searches and phylogenetic analyses were used to infer the best scenario. If paralogs had top BLAST hits to the same or a sister organism then these genes were assumed to be generated by one HGT event followed by duplication. Alternatively, paralogs from multiple transfer events had different putative donors located in different clades of the trees. In addition, these proteins were used to TBLASTN against the whole genome of *Bd* (*e* = 10^−10^) to identify potential pseudogenes, those that could not translate.

### Putative functional assignment

We performed COG, GO, and KEGG analyses for each horizontally transferred gene to infer its function. The KEGG analyses mainly investigated if proteins from HGT participated in metabolic networks of *Bd*s. We used KAAS (Kegg automated annotation server) to project the transferred genes onto Kegg's collection of metabolic pathways (Moriya et al., 2007) by BDH (bidirectional hit) orthology searching.

All HGT proteins were subjected to SignalP (Bendtsen et al., 2004), TargetP (Emanuelsson et al., 2007), and TMHMM (Sonnhammer et al., 1998) analyses to identify and predict their secreted proteins and to look for possible secretion signals and/or transmembrane domains. We also used WoLFPSORT (Horton et al., 2007) to investigate their putative cellular locations.

### Selection analyses

Selection pressure analyses were conducted for each HGT gene. We estimated the ML computation of non-synonymous (Ka) and synonymous (Ks) substitution rates, and their ratio Ka/Ks omega values for horizontally transferred genes in *Bd* and their homologs in corresponding donors. Codeml in the PAML 4 package was used to calculate the Ka/Ks values (Yang, 2007).

## Results

### Twenty-three gene families in two *Bd* strains via HGT

**Figure 2:**
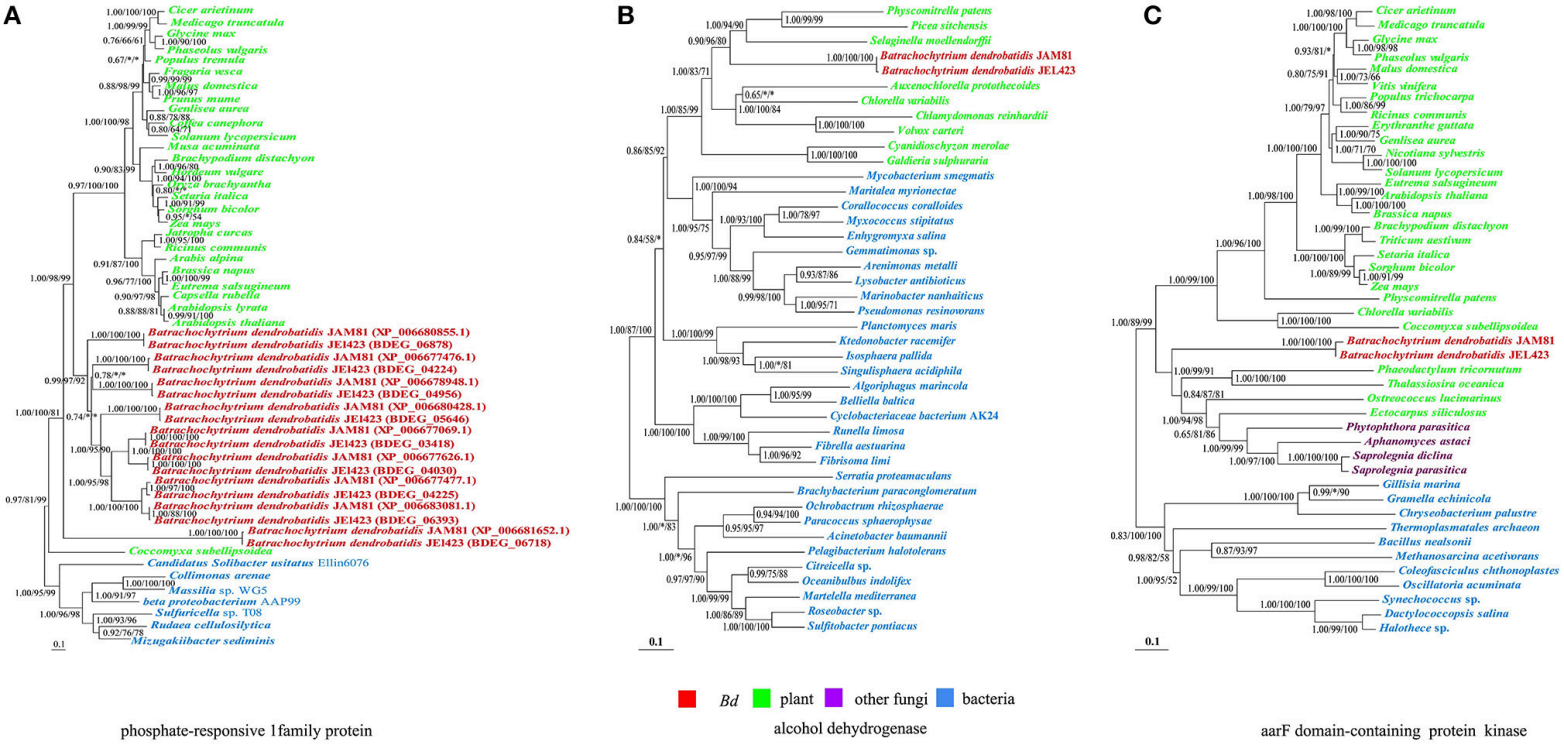
**Phylogenies of genes encoding (from left to right) phosphate-responsive 1 family protein, alcohol dehydrogenase and aarF domain-containing protein kinase horizontally transferred from plants to *Bd***. The Bayesian inference tree is shown unrooted. The Bayesian tree is virtually identical to ML and NJ trees. Numbers at nodes represent bayesian posterior probabilities **(A)** and bootstrap values of maximum likelihood **(B)** and neighbor-joining **(C)** respectively. Asterisks (^*^) indicate support values < 50. The scale bar corresponds to the estimated number of amino acid substitutions per site.

Stringent filters were employed to identify promising HGT genes in the annotated protein sequences of *Bd* JAM81 and *Bd* JEL423. Searches for protein-coding genes not broadly shared with other fungi yielded 4681 and 4884 homologs for *Bd* JAM81 and JEL423, respectively. No more than five fungal taxa shared these genes with *Bd*. Among these proteins, 737 and 879, respectively, were highly similar to those of non-fungi and, thus, they were considered to be candidate HGT genes. In addition to serine peptidases and CRN (Sun et al., 2011), phylogenetic analyses identified 54 and 50 proteins belonging to 23 families in *Bd* JAM81 and *Bd* JEL423, respectively, as being potentially obtained by HGT (Figure 1). All 23 of these families were validated by BI, ML phylogenies, as well as NJ trees, and with Bayesian posterior probabilities >90% and bootstrap support values of ML and NJ greater than 80% (Table 1, Figure 2, and Figure S1). The expressed data (Rosenblum et al., 2012) indicated all these candidate HGT genes were with expression, suggesting they were functional.

**Table 1:** **The 23 types of candidate HGT genes in *Bd* genome**.

**No**.	**Putative gene product**	**Putative donor**	***E*-value**	**Identity (%)**
1	Phosphate-responsive 1 family protein	Plant	8E-34	36
2	Alcohol dehydrogenase	Plant	5E-101	46
3	aarF domain-containing protein kinase	Plant	7E-33	33
4	Metallo-beta-lactamase	Bacteria	3E-38	39
5	Adenylate cyclase	Bacteria	3E-95	36
6	Carbonic anhydrase	Bacteria	2E-81	57
7	Chitinase	Bacteria	3E-99	50
8	Sel1 repeat-containing protein	Bacteria	3E-110	45
9	Secreted glycoside hydrolase	Bacteria	3E-80	42
10	3-mercaptopyruvate sulfurtransferase	Bacteria	2E-80	49
11	Arsenate reductase	Bacteria	1E-53	66
12	Membrane protein	Bacteria	3E-36	42
13	Alpha/Beta hydrolase	Bacteria	3E-55	33
14	Carboxylate-amine ligase	Bacteria	2E-35	33
15	Glutathione peroxidase	Bacteria	2E-71	63
16	Glutathione synthetase	Bacteria	7E-42	37
17	Aminopeptidase	Bacteria	7E-167	38
18	Glutamine synthetase	Bacteria	0	51
19	Succinylglutamate desuccinylase	Bacteria	1E-100	48
20	D-alanine–D-alanine ligase	Bacteria	8E-167	39
21	Glutamyl-tRNA amidotransferase	Bacteria	6E-15	48
22	Rhodanese-related sulfurtransferase	Bacteria	6E-29	57
23	Ankyrin repeat-containing protein	Oomycete	5E-113	51

The putative donors of transferred genes included prokaryotes, plants and oomycetes. *Bd* appeared to have derived three gene families that code for phosphate-responsive 1 family protein (Figure 2A), alcohol dehydrogenase (Figure 2B) and aarF domain-containing protein kinase from plants (Figure 2C). Their expression indicated that they are functional and not pseudogenes. In especial, to phosphate-responsive 1 family protein (Figure 2A), most plant species own this gene, indicating it is widely present in plant, while only a few fungi have this gene, so it is more possible that this gene in *Bd* is transferred from plant; while this gene in coccomyxa may be transferred from *Bd* or other fungi species. HGT events from plants to fungi were rare and only a few of these transfer events have been reported. Considering the sparse occurrence, these three gene families derived from plants may endow the recipient *Bd* with novel important traits and have important role in evolution and adaptation of *Bd*. Other 19 gene families were acquired from various bacteria. The aquatic habitat of some bacterial donors may have facilitated the transfer of genetic material from them to *Bd*. In addition, *Bd* also obtained one ankyrin repeat-containing protein family from oomycete pathogens. This gene subsequently duplicated in *Bd*. All these acquired genes have no endogenous homologs in *Bd*, indicating that they offer novel functions to *Bd*.

We also explored possible HGT between *Bd* and their amphibian hosts but without success. Several protein sequences of *Bd* that were missing from other fungi had high similarity with their amphibian hosts. However, phylogenetic analyses did not support the hypothesis of HGT from amphibians to *Bd*.

Several lines of evidence eliminate the possibility of contamination from bacteria, oomycetes or plants, which could explain our results. First, all transferred genes occur in both *Bd* strains, rendering contamination unlikely (Richards et al., 2011b). Second, contigs having the horizontally transferred genes contain more than five genes total, thus assuring quality of the assemblies and further precluding contamination. In addition, all flanking DNA sequences of the transferred genes show substantial similarity to fungal sequences. Fungal sequences of vertical inheritance surround the putatively transferred genes. Thus, native fungal sequences link to horizontally transferred genes (Richards et al., 2011b). Finally, most of transferred genes contain introns (Table S2), which further precludes bacterial contaminates.

### Multi-copies of 10 horizontally transferred gene families in *Bd*

**Figure 3:**
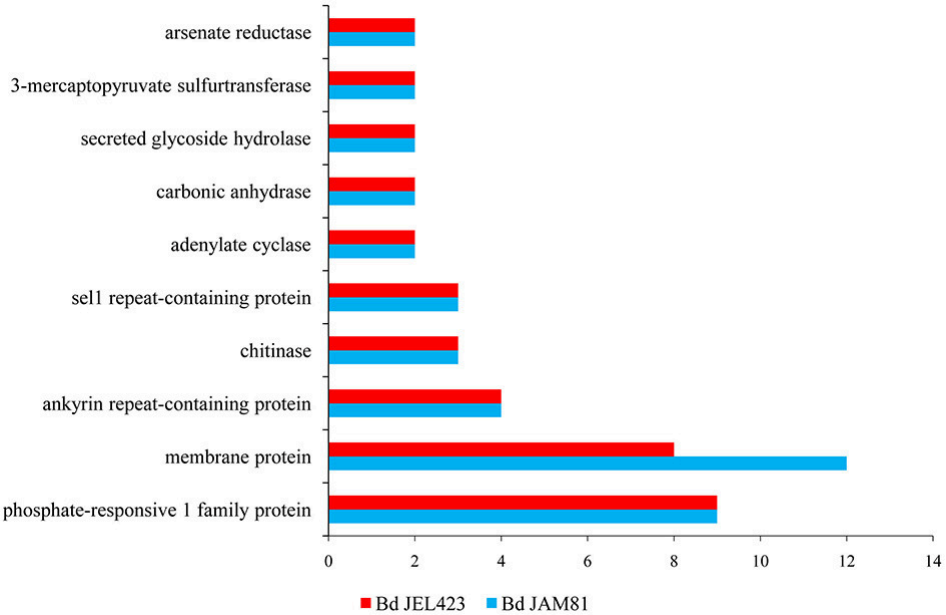
**The copy number of horizontally transferred genes with paralogs in the two strains of *Bd***.

Both serine peptidases and CRN proteins were indicated to be highly duplicated in *Bd* (Sun et al., 2011). Among our 23 newly discovered horizontally transferred gene families, phosphate-responsive 1 family protein, membrane protein, ankyrin repeat-containing protein, chitinase, sel1 repeat-containing protein, adenylate cyclase, carbonate dehydratase, secreted glycoside hydrolase, 3-mercaptopyruvate sulfurtransferase, and arsenate reductase harbored a diverse number of copies (Figure 3). On the one hand, adenylate cyclase and carbonic anhydrase appeared to have undergone multiple HGT events for their copies located in different branches near potential donor species (Figure S1). On the other hand, some gene families diversified via duplication subsequent to a single HGT event, for their copies cluster together as the sister-clade to the donors (Figure S1), which indicated duplications (Nikoh et al., 2010). For example, both *Bd* strains had nine copies of the gene family encoding the phosphate-responsive 1 family protein, all of which constituted the sister-clade of plant homologs (Figure 2A). A similar evolutionary scenario occurred for the gene families encoding membrane proteins, all of the gene copies clustered with bacterial sequences (Figure S1).

### Diverse metabolic functions of transferred genes

The putative function and the biochemical pathways of the HGT candidates were deduced with COG, GO, and KEGG and are summarized in Table 2. COG categories indicated that these genes were mainly involved in energy production and conversion, signal transduction mechanisms, carbohydrate, amino acid, and inorganic ion transport and metabolism. In addition, some genes related functionally to cell wall/membrane/envelope biogenesis, posttranslational modification, protein turnover and chaperones. GO analysis indicated that their functions related to a variety of activities, such as catalytic, hydrolase, transferase, cyclase, ligase, lyase, and oxidoreductase activities. Furthermore, some genes associated with antioxidant, peroxidase, arsenate reductase, responses to hypoxia, mechanical stimulus and growth. Most transferred genes had multiple functions and some functions appeared to benefit *Bd* considerably. These gene functions associated importantly with its niche adaptation. Functions such as antioxidant, peroxidase, and arsenate reductase activities likely related to its stress responses. In the KEGG databases, most genes appeared to be involved in sugar, carbohydrate, amino acid biosynthesis, and metabolism. Some genes participated in aspartate, glutamate, glutathione, arachidonic acid, glyoxylate and dicarboxylate, nitrogen and purine metabolism, which are critical for its life. Several independently transferred gene families participated in the same metabolic pathways, such as glutathione peroxidase, glutathione synthetase, and aminopeptidase, all of which related to glutathione metabolism. Overall, function analyses indicated these transferred genes endowed abundant novel functions to *Bd*.

**Table 2:** **The COG, GO, and KEGG biochemical pathway mappings and the predicted cellular locations of HGT genes**.

**No**.	**Gene name**	**COG categories**	**GO categories**	**KEGG pathways**	**TargetP**	**SignalP**	**WoLF PSORT**
1	Phosphate-responsive 1 family protein	Unknown	Microtubule nucleation	Unknown	S	YES	**extr**
2	Alcohol dehydrogenase	Energy production and conversion	Alcohol dehydrogenase activity	Metabolic pathways	–		cyto
3	aarF domain-containing protein kinase	Unknown	ATP binding protein amino acid phosphorylation	Unknown	–		cyto
4	Metallo-beta-lactamase	Predicted sugar phosphate isomerase	Beta-lactamase activity	Unknown	–		cyto
5	Adenylate cyclase	Signal transduction mechanisms	Guanylate cyclase activity intracellular signaling cascade	Purine metabolism	M		mito
				Nucleotide metabolism			
6	Carbonic anhydrase	Inorganic ion transport and metabolism	Carbon utilization	Nitrogen metabolism	–		nucl
7	Chitinase	Carbohydrate transport and metabolism	Catalytic activity hydrolase activity	Carbohydrate metabolism	S	YES	**extr**
8	Sel1 repeat-containing protein	Cell wall/membrane/envelope biogenesis signal transduction mechanisms	Unknown	Unknown	M		cyto
9	Secreted glycoside hydrolase	Unknown	Glucosylceramidase activity hydrolase activity	Carbohydrate metabolic process	S		**extr**
10	3-mercaptopyruvate sulfurtransferase	Inorganic ion transport and metabolism	Catalytic activity transferase activity	Cysteine and methionine metabolism	M		cyto
11	Arsenate reductase	Signal transduction mechanisms	Tyrosine phosphatase activity arsenate reductase activity	Unknown	–		cyto
12	Membrane protein	Unknown	Integral to membrane	Unknown	–		plas
13	Alpha/Beta hydrolase	Unknown	Hydrolase activity catalytic activity	Unknown	S		**extr**
14	Carboxylate-amine ligase	Unknown	Catalytic activity ligase activity	Peptidoglycan biosynthetic process	–		cyto
15	Glutathione peroxidase	Posttranslational modification protein turnover, chaperones	Response to oxidative stress antioxidant activity	Glutathione metabolism	–		cyto
				Arachidonic acid metabolism			
16	Glutathione synthetase	Unknown	Glutathione synthase activity glutathione biosynthetic process	Glutathione metabolism	–		cyto
17	Aminopeptidase	Amino acid transport and metabolism	Proteolysis and peptidolysis aminopeptidase activity	Glutathione metabolism	–		cyto
18	Glutamine synthetase	Amino acid transport and metabolism	Glutamate-ammonia ligase activity nitrogen fixation	Alanine, aspartate and Glutamate metabolism	M		mito
19	Succinylglutamate desuccinylase	Unknown	Hydrolase activity catalytic activity	Unknown	–		cyto
20	D-alanine–D-alanine ligase	Cell wall/membrane/envelope biogenesis	S-adenosylmethionine-dependent methyltransferase activity	D-Alanine metabolism	–		cyto
				Peptidoglycan biosynthesis			
21	Glutamyl-tRNA amidotransferase	Unknown	Carbon-nitrogen ligase activity	Unknown	M		mito
22	Rhodanese-related sulfurtransferase	Unknown	Transferase activity	Sulfur relay system	–		cyto
				Sulfur metabolism			
23	Ankyrin repeat-containing protein	Unknown	Unknown	Unknown	S		**extr**

*The genes without definite information in the database were indicated with unknown. For genes participating in multiple function and biochemical pathways, all of them were shown. In the sixth column, S means secretory pathway, M refers to mitochondrion location and the minus means any other location. In the seventh column, YES means that the sequence has signal peptide. In the eighth column, extr, cyto, and mito show that the protein is likely localized in extracellular sites (highlighted in bold font), cytoplasm and mitochondria, respectively*.

**Figure 4:**
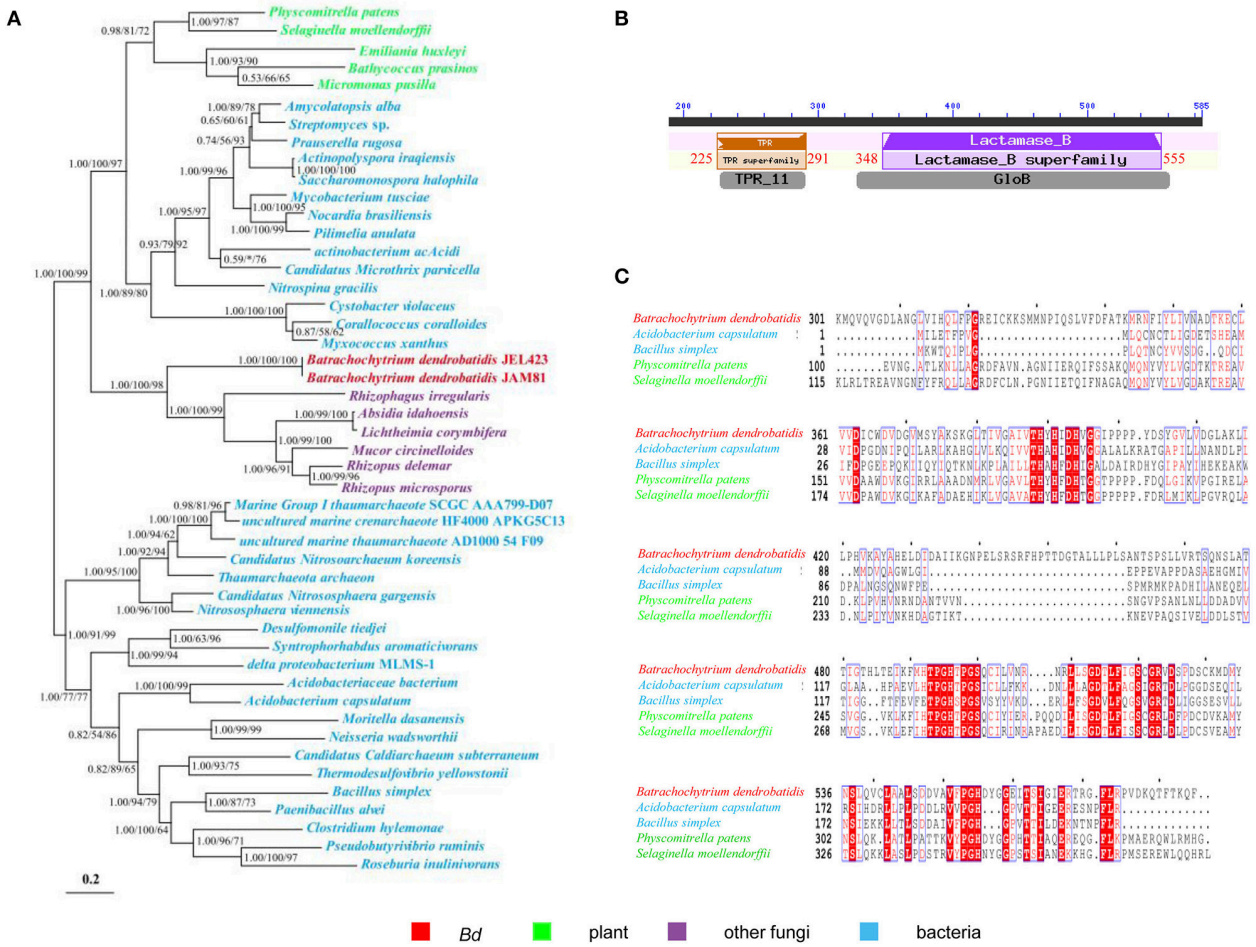
**The phylogeny and domain of the gene encoding metallo-beta-lactamase for *Bd* and other organisms. (A)** Phylogeny showing HGT from bacteria to *Bd*. The Bayesian tree (shown) is virtually identical to ML and NJ trees. Nodal support values ≥50 shown (BI/ML/NJ). Asterisks (^*^) indicate support values < 50. Scale bar indicates substitutions per site. **(B)** Conserved domain of TRP (Tetratricopeptide repeat domain, Location: 225–291 nt) and Lactamase_B (Metallo-beta-lactamase superfamily, Location: 348–555 nt) in the gene of *Bd* encoding metallo-beta-lactamase. **(C)** Alignment of the gene of *Bd* encoding metallo-beta-lactamase and other organisms. Only the aligned regions are shown.

The acquisition of metallo-beta-lactamase and arsenate reductase from bacteria to *Bd* may have promoted defense. Metallo-beta-lactamases confer resistance to a broad range of antibiotics and inhibitors. Its HGT likely plays a major role in conquering new environments and hosts. The transfer of this gene from bacteria to fungi has been reported rarely. To *Bd*, its metallo-beta-lactamase well clusters within bacteria (Figure 4A) and contains conserved Lactamase_B domains as plant and bacteria (Figures 4B,C). Such transfers may play great roles in adaptation of *Bd*. The arsenate reductase is essential for arsenic detoxification and resistance by transforming arsenate into arsenite. Thus, the acquisition of a bacterial arsenate reductase by *Bd* might have endowed them with the ability to detoxify arsenics (Marcet-Houben and Gabaldón, 2010).

### Putative cellular locations of HGTs

Five horizontally transferred gene families have presumed extracellular locations. These include phosphate-responsive 1 family protein, chitinase, secreted glycoside hydrolase, alpha/beta hydrolase and ankyrin repeat-containing protein (Table 2). Extracellular enzymes may relate to interactions between *Bd* and its hosts. Among these gene families, phosphate-responsive 1 family protein, which was probably derived from plant, relates to responses to hypoxia and mechanical stimulus as well as growth in plant. Its subsequent duplication in *Bd* implies important function(s). Chitinase, secreted glycoside hydrolase and alpha/beta hydrolase involve hydrolase activity, which may relate to the efficient infection of hosts by *Bd*. These enzymes have been reported to regulate the interactions between pathogenic bacteria and their hosts (Zhu et al., 2012), indicating an importance roles in adaptation of *Bd*. The bacterial origin and extracellular location of these enzymes in *Bd* suggests they may serve to break down chitin, which in amphibians serves to trigger an allergy/immune response (Tang et al., 2015). The duplication of genes encoding ankyrin repeat-containing protein (ANK) derived from oomycete indicates a great role played by this HGT gene family. ANK proteins were reported to represent a new family of bacterial type IV effectors that play a major role in host-pathogen interactions and the evolution of infections (Siozios et al., 2013). Thus, these proteins in *Bd* may also relate to host-pathogen interactions. Other HGT gene families that encode intracellular proteins play roles in various metabolic activities and their acquisitions relates to functional requirements of the recipients, highly utilization of carbohydrates and nutrition. Thus, the acquisition of multiple genes by HGT endows the recipient *Bd* with great advantages in its evolution and adaption.

### Purifying selection of horizontally transferred genes

To assess selective pressure of these HGT genes, we computed the synonymous (Ks) and non-synonymous (Ka) substitution rates, and the ratio Ka/Ks for all these HGT genes. Generally, Ka/Ks < 1 was assumed to indicate purifying selection, Ka/Ks = 1 indicated neutral evolution, and Ka/Ks > 1 indicated positive selection. Ka/Ks values for all transferred genes are less than 0.5, suggesting that they were undergone purifying selection. In general, Ka/Ks values relate to protein function and low values indicate that selective constraints has acted on functional genes, so the demonstration that selective constraints are operating on these genes supports the conclusion that these genes are fully functional in *Bd* (Wu and Zhang, 2011).

## Discussion

In this study, we used a semi-automated pipeline to identify HGT candidates in *Bd*. The construction and analysis of phylogenetic trees is recognized as the most reliable method for detecting HGT, so we included several steps of manual phylogenetic trees inspection in the pipeline. Using this pipeline, we detected 3 gene families with evidences of HGT from plant, 19 HGTs from various bacteria and one from oomycete. HGT from plants to fungi is reported rarely. To data, only few evidences of these transfer events have been reported: UDP-glucosyltransferase in *Botrytis cinerea* was derived from *Hieracium pilosella*, one leucine-rich repeat protein in *Pyrenophora* was transferred from host barley and four additional plant-to-fungi HGTs are known (Richards et al., 2009; Zhu et al., 2012; Sun et al., 2013). Here, we provided three well evidences of plant-fungi transfer events in *Bd*. In addition, it is well known that *Bd* is an amphibian parasitic fungi, HGT from plants to animal pathogenic fungi is fascinating. The aquatic environment provides an opportunity for *Bd* to obtain genes from aquatic plants. Among these three plant-derived gene families, alcohol dehydrogenase transferred between different bacteria and from bacteria to protists and fungi has been described (Field et al., 2000; Trcek and Matsushita, 2013). In contrast, its transfer from plants to fungi is rarely known. Functioning in protein kinase activity, aarF domain-containing protein kinase participates in protein amino acid phosphorylation. Further, phosphate-responsive 1 family protein duplicated subsequent to the HGT event. Their function annotations and evolution events indicated that these three plant-derived gene families may endow the recipient *Bd* with novel traits and enhance the adaptation of *Bd* to its hosts.

Bacteria and plants can donate genes to fungi via inter-kingdom HGT and so can animals. The gene encoding purine nucleotide phosphorylase (PNP) in *Encephalitozoon romaleae* was transferred from its insect host (Selman et al., 2011), as was the sterol carrier gene in *Metarhizium robertsii* and the latter transfer facilitated the pathogen's infection of insects (Zhao et al., 2014). In comparison, no evidence supports the transfer of genes from amphibian hosts to *Bd*. Nevertheless, we detected some proteins of *Bd* that were missing from other fungi had high similarity with their amphibian hosts, but the phylogenetic trees did not support the HGT hypothesis for they did not cluster with the amphibian hosts well. In our opinion, their high similarity with amphibian hosts may reflect molecular mimicry. This mechanism can allow fungal pathogens to mimic the proteins of their vertebrate hosts to evade the immune responses, such as occurs in parasitic nematodes (Shinya et al., 2013). Future functional experiments can further verify our speculation.

It is possible that the candidate HGT genes were in the common ancestor of donors and fungi but were lost subsequently in many fungi. However, this explanation is highly unlikely for three reasons. First, it requires massive independent losses to explain the presence of these genes only in *Bd* and a few other fungi only. Second, this hypothesis fails to explain the remarkable sequence identity between *Bd* and its candidate donors. Finally, loss fails to explain the clustering of HGT genes within bacteria, oomycetes or plants on well-supported nodes of the gene trees.

Duplications of some HGT genes further suggested their significance to *Bd*. The timing of HGT genes' duplications varies widely. Genes involved in membrane proteins appear to be recent duplications as indicated by the high levels of similarity among their copies, many of which show very little sequence variation. In contrast, the duplication of the phosphate-responsive 1 family protein exhibits a low level of similarity among its nine copies. These appear to be relatively ancient duplications. Most gene duplications events predate divergence of the two strains of *Bd*. Furthermore, neither *Bd* genome has pseudogenes among the 23 families. Gene duplication can contribute to the expansion of protein families (Danchin et al., 2010). The generation of new copies may relate to the specialized life and functional requirements of *Bd*.

Functional annotations and cellular locations indicate that the horizontally transferred genes not only participate in infection process, but also take part in various metabolic progresses. So, the acquisition of these genes contributes to evolution and adaption of *Bd* significantly. They can confer new capabilities to the recipient *Bd*: highly efficient infections of hosts and utilization of host carbohydrates and nutrition.

Moreover, it is meaningful that some HGT genes participate in same pathways. The acquisitions of multiple genes by HGT that participate in concert in same metabolic pathways have greater significance to the recipients than those that work alone. In fungi, glutathione plays key roles in their response to several stressful situations. Organisms mobilize glutathione under sulfur or nitrogen starvation to ensure cellular maintenance. Moreover, induction of the glutathione-dependent antioxidative defense system in response to carbon deprivation stress results in an increased tolerance to oxidative stress. This confers resistance against heat shock and osmotic stress, indicating that transfers of three genes participating in this pathway is important given *Bd*'s aquatic lifestyle (Pócsi et al., 2004).

## Conclusion

In summary, our results illustrate the scale of HGT and the roles it plays in the rapid evolution and adaptation of *Bd*. These horizontally transferred genes may be key adaptations that enhance colonization on amphibian hosts, perhaps leading to more effective spreading and competitive advantage. Meanwhile, these HGTs event may endow important evolutionary implications for *Bb* and its diseases in general. Future experiments will enable a better understanding of the functions and contributions of these HGTs to *Bd*.

## Author contributions

Conceived and designed the experiments: BS, TL, and DH. Analyzed the data: BS, TL, and JX. Contributed reagents/materials/analysis tools: LL, PZ, and SH. Wrote the paper: BS, TL, RM, and DH.

### Conflict of interest statement

The authors declare that the research was conducted in the absence of any commercial or financial relationships that could be construed as a potential conflict of interest.

## Supplementary material

Figure S1 **Phylogenetic analyses of horizontally transferred genes in *Bd* derived from bacteria and oomycete**. The Bayesian inference tree is shown unrooted. The Bayesian tree is virtually identical to ML and NJ trees. Numbers at nodes represent bayesian posterior probabilities (left) and bootstrap values of maximum likelihood (middle) and neighbor-joining (right) respectively. Asterisks (^*^) indicate support values < 50. The scale bar corresponds to the estimated number of amino acid substitutions per site.

Table S1 **The code (script) and parameters used in the bioinformatic pipeline**.

Table S2 **Exon number for each horizontally transferred gene in the two strains of *Bd***.
