# Supplementary material for: Contribution of Multiple Inter-Kingdom Horizontal Gene Transfers to Evolution and Adaptation of Amphibian-Killing Chytrid, Batrachochytrium dendrobatidis
Source: Front Microbiol. 2016 Aug 31;7:1360. doi: 10.3389/fmicb.2016.01360 (PMC5005798; doi:10.3389/fmicb.2016.01360)
Supplement: Figure S1 — Phylogenetic analyses of horizontally transferred genes in Bd derived from bacteria and oomycete. The Bayesian inference tree is shown unrooted. The Bayesian tree is virtually identical to ML and NJ trees. Numbers at nodes represent bayesian posterior probabilities (left) and bootstrap values of maximum likelihood (middle) and neighbor-joining (right) respectively. Asterisks (*) indicate support values < 50. The scale bar corresponds to the estimated number of amino acid substitutions per site. [file Image1.PDF]

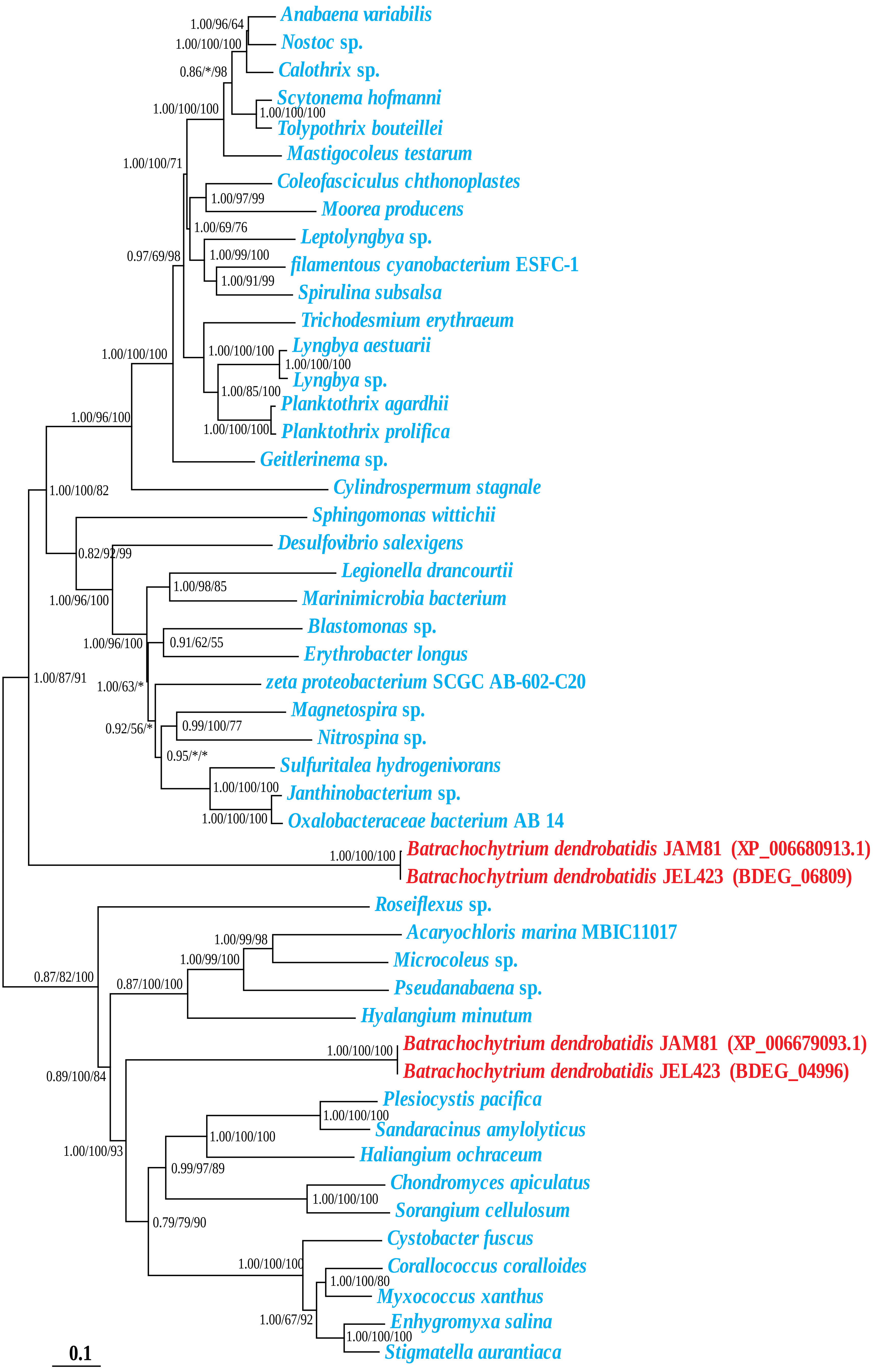

adenylate cyclase

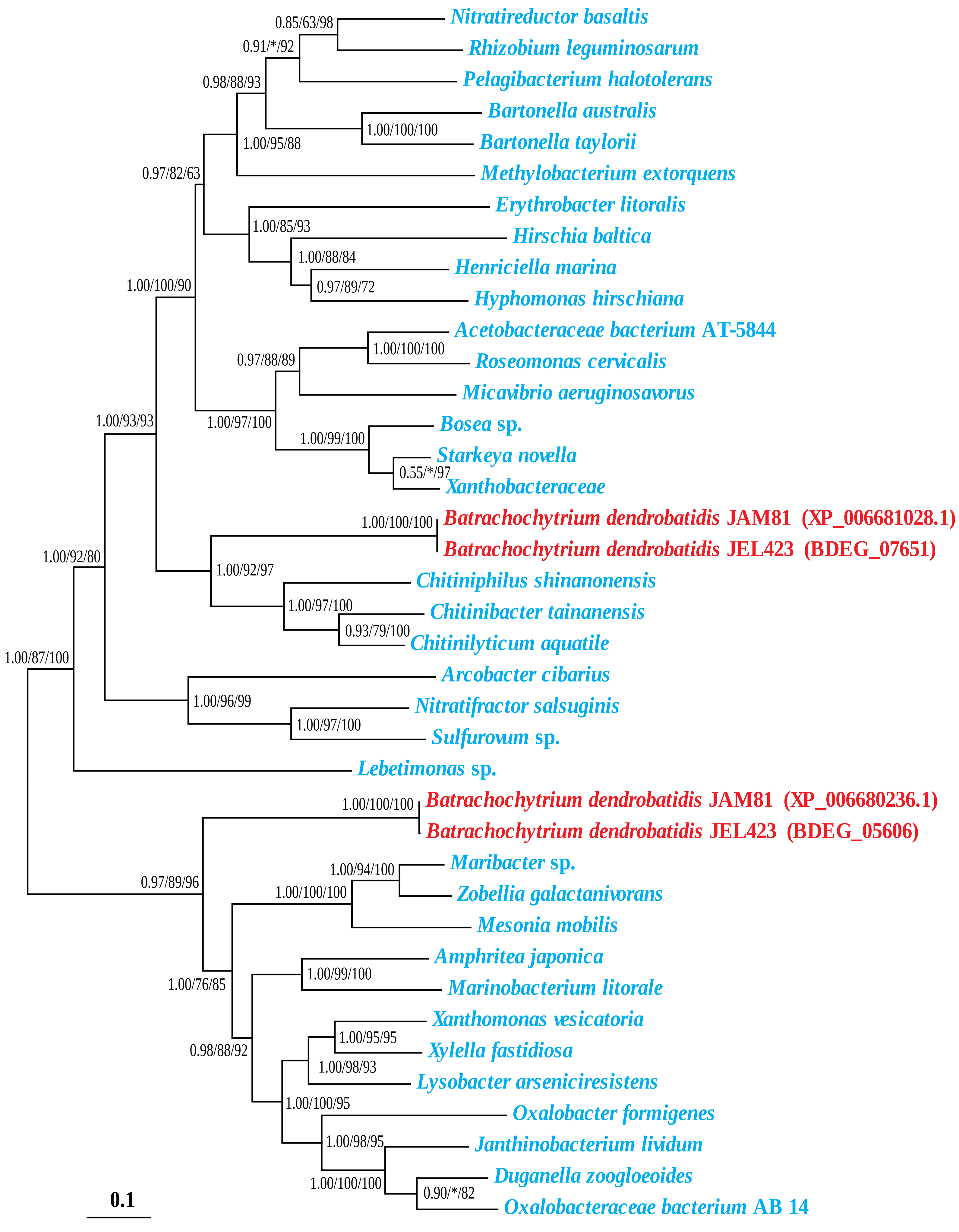

carbonic anhydrase

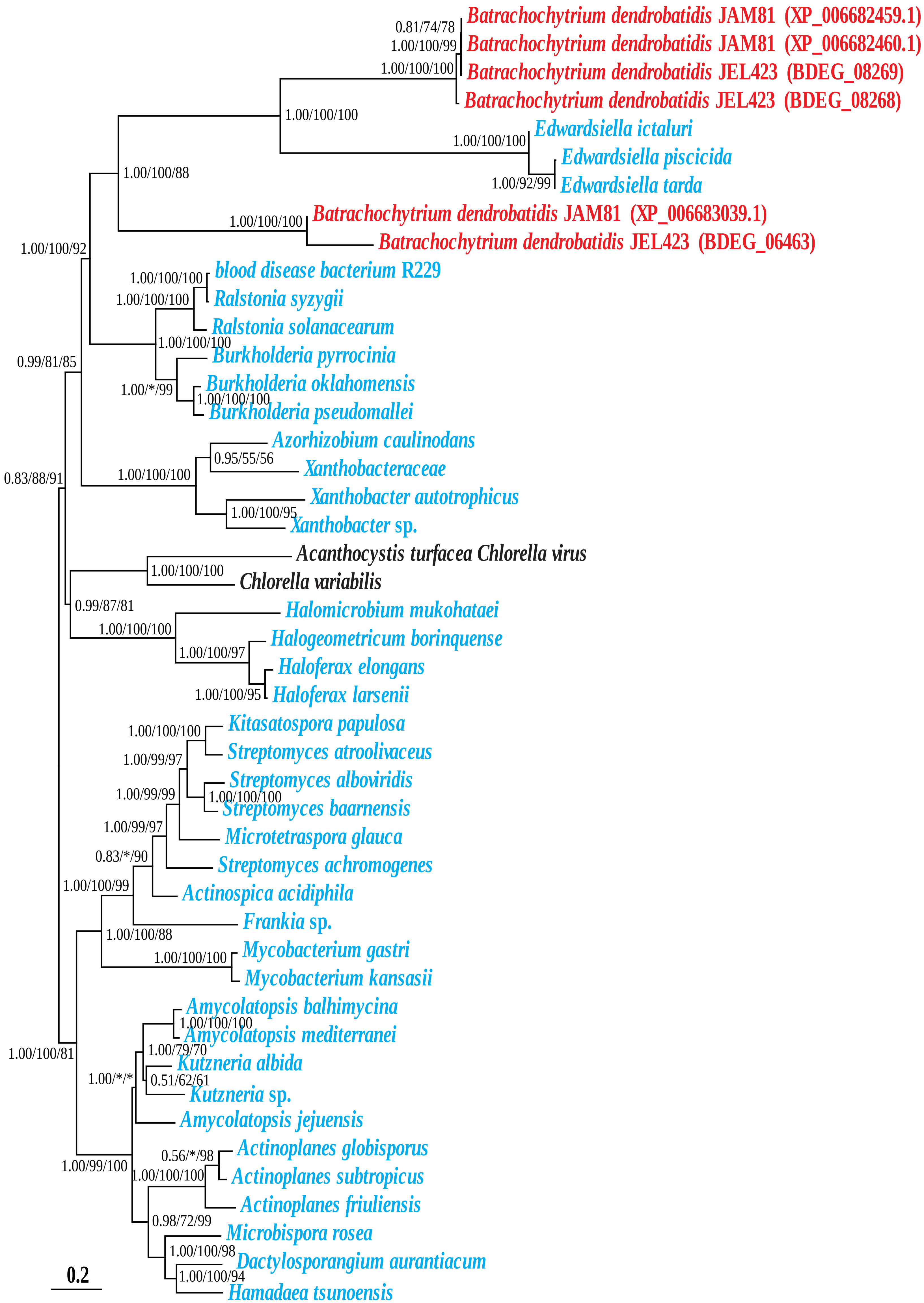
Batrachochytrium dendrobatidis JAM81 (XP_006682459.1)
Batrachochytrium dendrobatidis JAM81 (XP_006682460.1)
Batrachochytrium dendrobatidis JEL423 (BDEG_08269)
Batrachochytrium dendrobatidis JEL423 (BDEG_08268)
Edwardsiella ictaluri
Edwardsiella piscicida
Edwardsiella tarda
Batrachochytrium dendrobatidis JAM81 (XP_006683039.1)
Batrachochytrium dendrobatidis JEL423 (BDEG_06463)
blood disease bacterium R229
Ralstonia syzygii
Ralstonia solanacearum
Burkholderia pyrrocinia
Burkholderia oklahomensis
Burkholderia pseudomallei
Azorhizobium caulinodans
Xanthobacteraceae
Xanthobacter autotrophicus
Xanthobacter sp.
Acanthocystis turfacea Chlorella virus
Chlorella variabilis
Halomicrobium mukohataei
Halogeometricum borinquense
Haloferax elongans
Haloferax larsenii
Kitasatospora papulosa
Streptomyces atroolivaceus
Streptomyces alboviridis
Streptomyces baarnensis
Microtetraspora glauca
Streptomyces achromogenes
Actinospica acidiphila
Frankia sp.
Mycobacterium gastri
Mycobacterium kansasii
Amycolatopsis balhimycina
Amycolatopsis mediterranei
Kutzneria albida
Kutzneria sp.
Amycolatopsis jejuensis
Actinoplanes globisporus
Actinoplanes subtropicus
Actinoplanes friuliensis
Microbispora rosea
Dactylosporangium aurantiacum
Hamadaea tsunoensis
0.81/74/78
1.00/100/99
1.00/100/100
1.00/100/100
1.00/100/100
1.00/92/99
1.00/100/88
1.00/100/100
1.00/100/92
1.00/100/100
1.00/100/100
1.00/100/100
0.99/81/85
1.00/*/99
1.00/100/100
0.95/55/56
0.83/88/91
1.00/100/100
1.00/100/95
1.00/100/100
0.99/87/81
1.00/100/100
1.00/100/97
1.00/100/95
1.00/100/100
1.00/99/97
1.00/99/99
1.00/100/100
1.00/99/97
0.83/*/90
1.00/100/99
1.00/100/88
1.00/100/100
1.00/100/100
1.00/79/70
1.00/100/81
1.00/*/*
0.51/62/61
0.56/*/98
1.00/100/100
1.00/99/100
0.98/72/99
1.00/100/98
1.00/100/94
0.2

chitinase

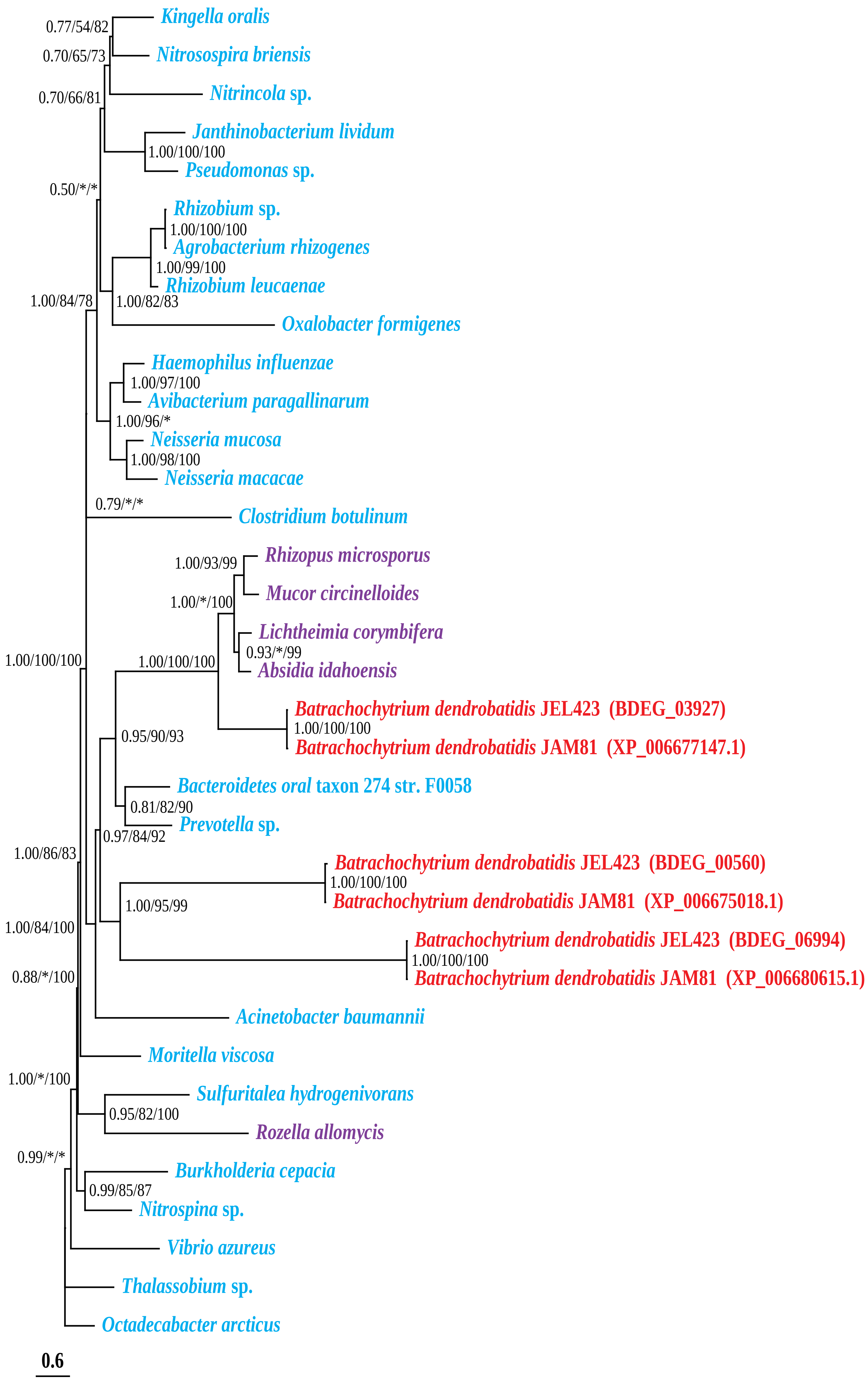

sel1 repeat-containing protein

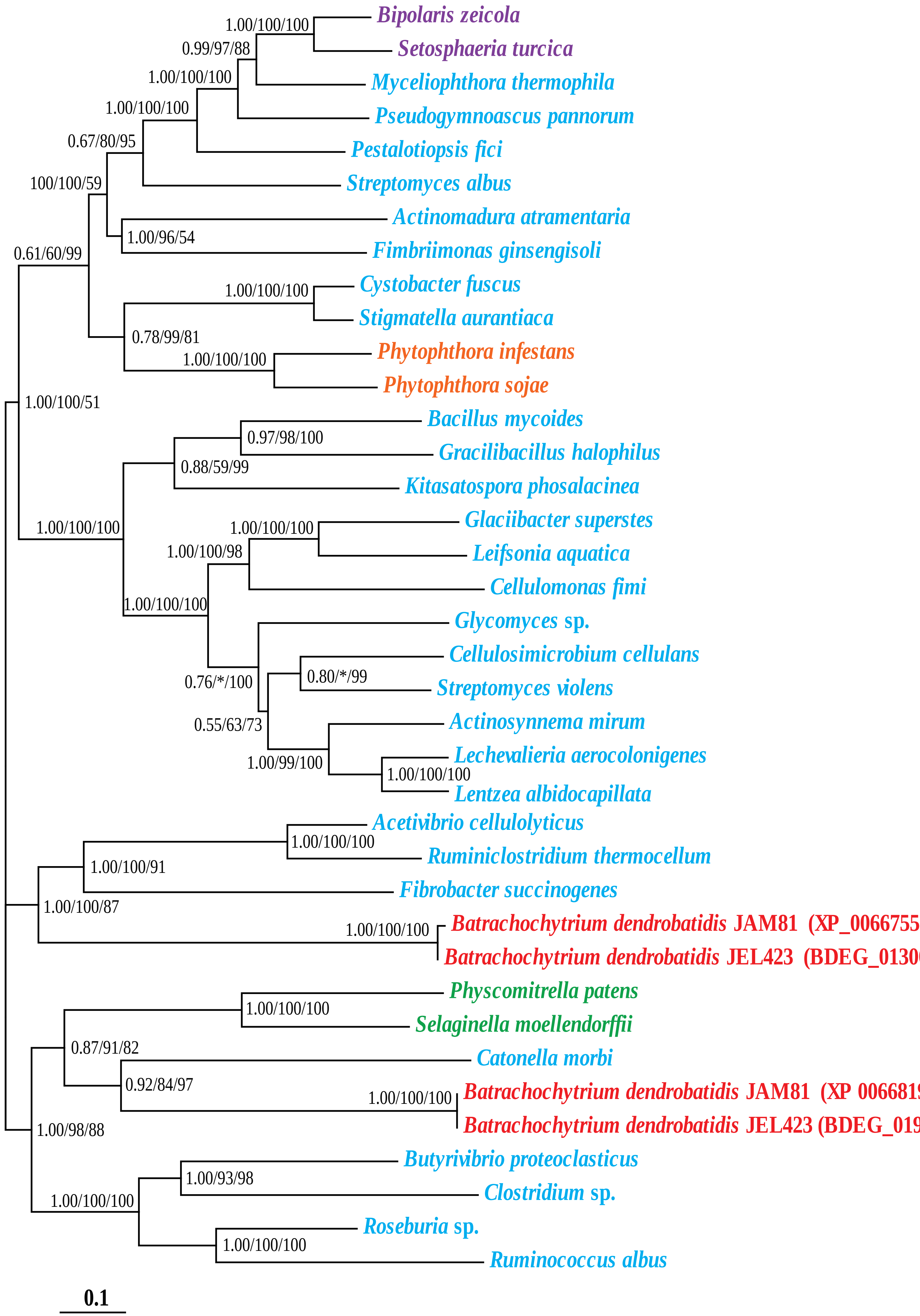

secreted glycoside hydrolase

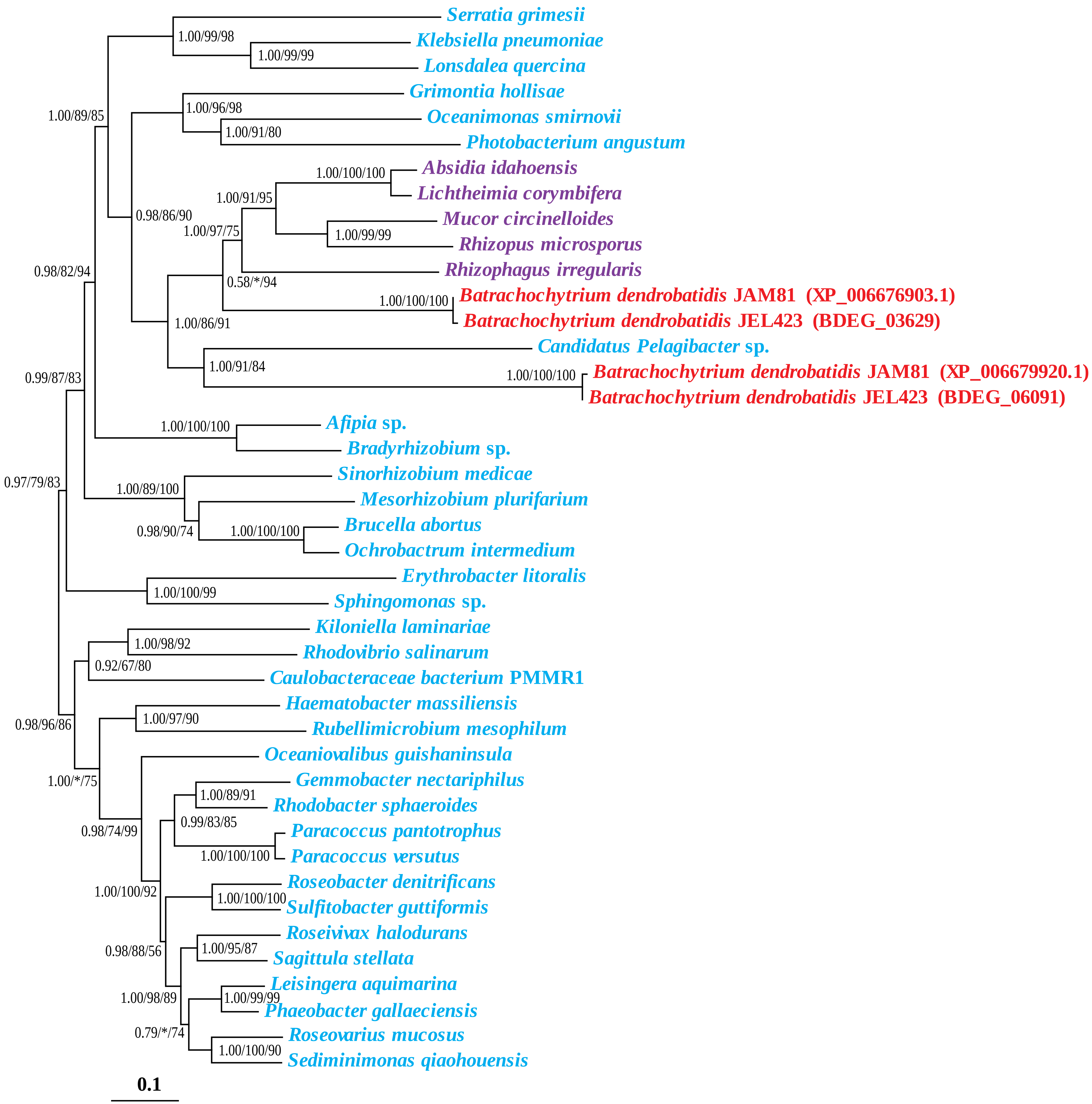

3-mercaptopyruvate sulfurtransferase

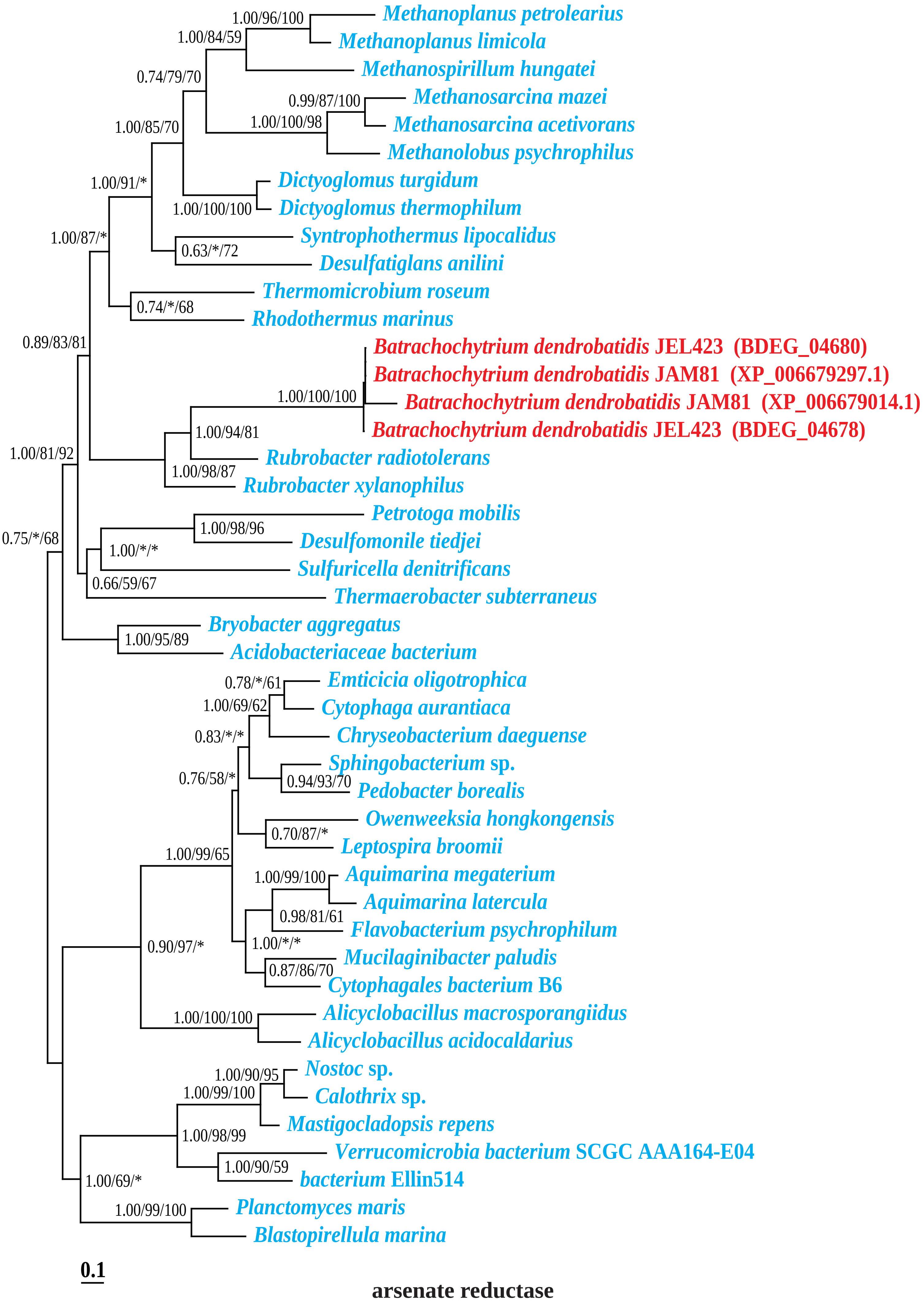

arsenate reductase

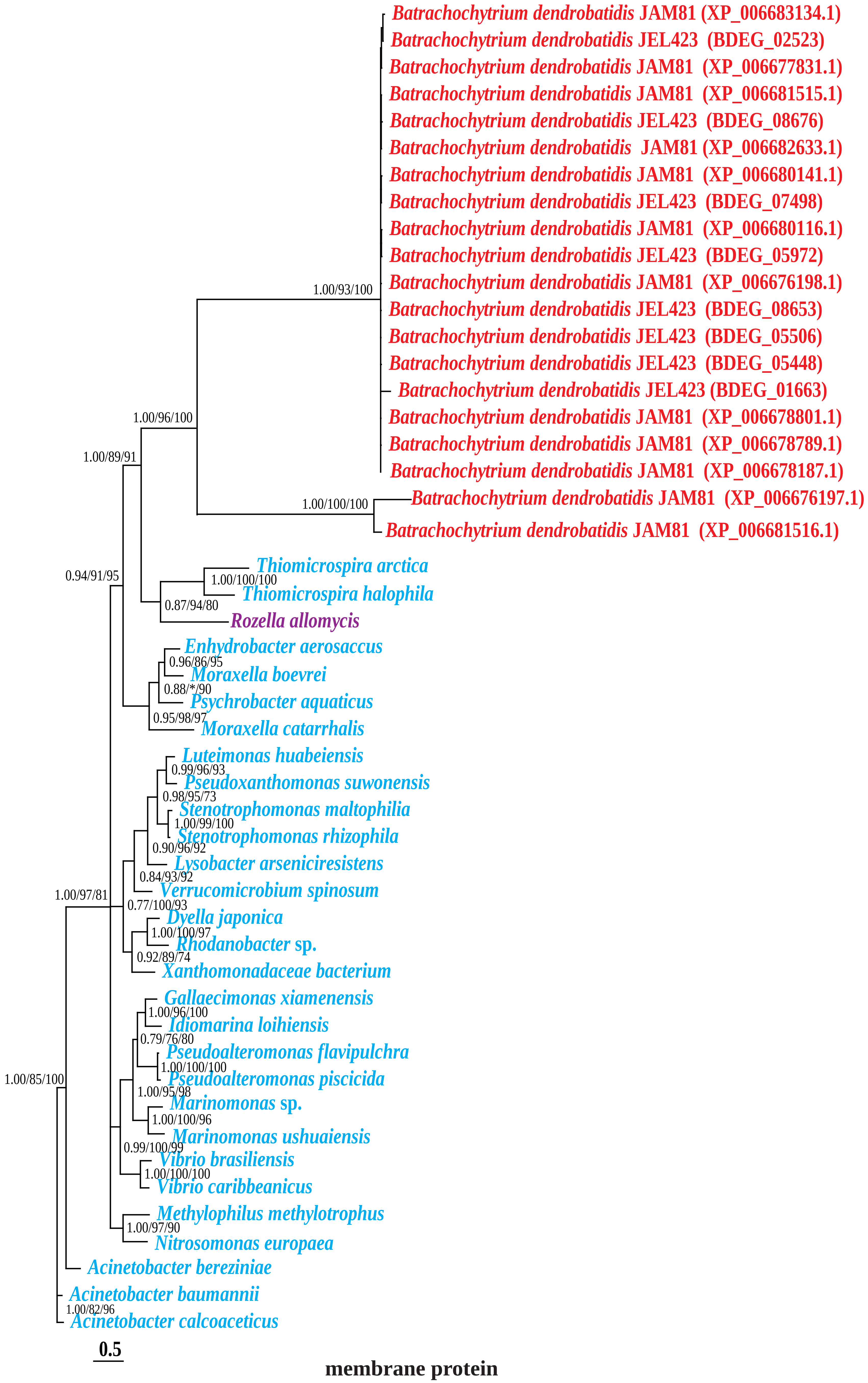
Batrachochytrium dendrobatidis JAM81 (XP_006683134.1)
Batrachochytrium dendrobatidis JEL423 (BDEG_02523)
Batrachochytrium dendrobatidis JAM81 (XP_006677831.1)
Batrachochytrium dendrobatidis JAM81 (XP_006681515.1)
Batrachochytrium dendrobatidis JEL423 (BDEG_08676)
Batrachochytrium dendrobatidis JAM81 (XP_006682633.1)
Batrachochytrium dendrobatidis JAM81 (XP_006680141.1)
Batrachochytrium dendrobatidis JEL423 (BDEG_07498)
Batrachochytrium dendrobatidis JAM81 (XP_006680116.1)
Batrachochytrium dendrobatidis JEL423 (BDEG_05972)
Batrachochytrium dendrobatidis JAM81 (XP_006676198.1)
Batrachochytrium dendrobatidis JEL423 (BDEG_08653)
Batrachochytrium dendrobatidis JEL423 (BDEG_05506)
Batrachochytrium dendrobatidis JEL423 (BDEG_05448)
Batrachochytrium dendrobatidis JEL423 (BDEG_01663)
Batrachochytrium dendrobatidis JAM81 (XP_006678801.1)
Batrachochytrium dendrobatidis JAM81 (XP_006678789.1)
Batrachochytrium dendrobatidis JAM81 (XP_006678187.1)
1.00/93/100
1.00/96/100
1.00/89/91
1.00/100/100
Batrachochytrium dendrobatidis JAM81 (XP_006676197.1)
Batrachochytrium dendrobatidis JAM81 (XP_006681516.1)
0.94/91/95
Thiomicrospira arctica
1.00/100/100
Thiomicrospira halophila
0.87/94/80
Rozella allomycis
Enhydrobacter aerosaccus
0.96/86/95
Moraxella boevrei
0.88/*/90
Psychrobacter aquaticus
0.95/98/97
Moraxella catarrhalis
Luteimonas huabeiensis
0.99/96/93
Pseudoxanthomonas suwonensis
0.98/95/73
Stenotrophomonas maltophilia
1.00/99/100
Stenotrophomonas rhizophila
0.90/96/92
Lysobacter arseniciresistens
0.84/93/92
Verrucomicrobium spinosum
1.00/97/81
0.77/100/93
Dyella japonica
1.00/100/97
Rhodanobacter sp.
0.92/89/74
Xanthomonadaceae bacterium
Gallaecimonas xiamenensis
1.00/96/100
Idiomarina loihiensis
0.79/76/80
Pseudoalteromonas flavipulchra
1.00/100/100
Pseudoalteromonas piscicida
1.00/85/100
1.00/95/98
Marinomonas sp.
1.00/100/96
Marinomonas ushuaiensis
0.99/100/99
Vibrio brasiliensis
1.00/100/100
Vibrio caribbeanicus
Methylophilus methylotrophus
1.00/97/90
Nitrosomonas europaea
Acinetobacter bereziniae
Acinetobacter baumannii
1.00/82/96
Acinetobacter calcoaceticus
0.5
membrane protein

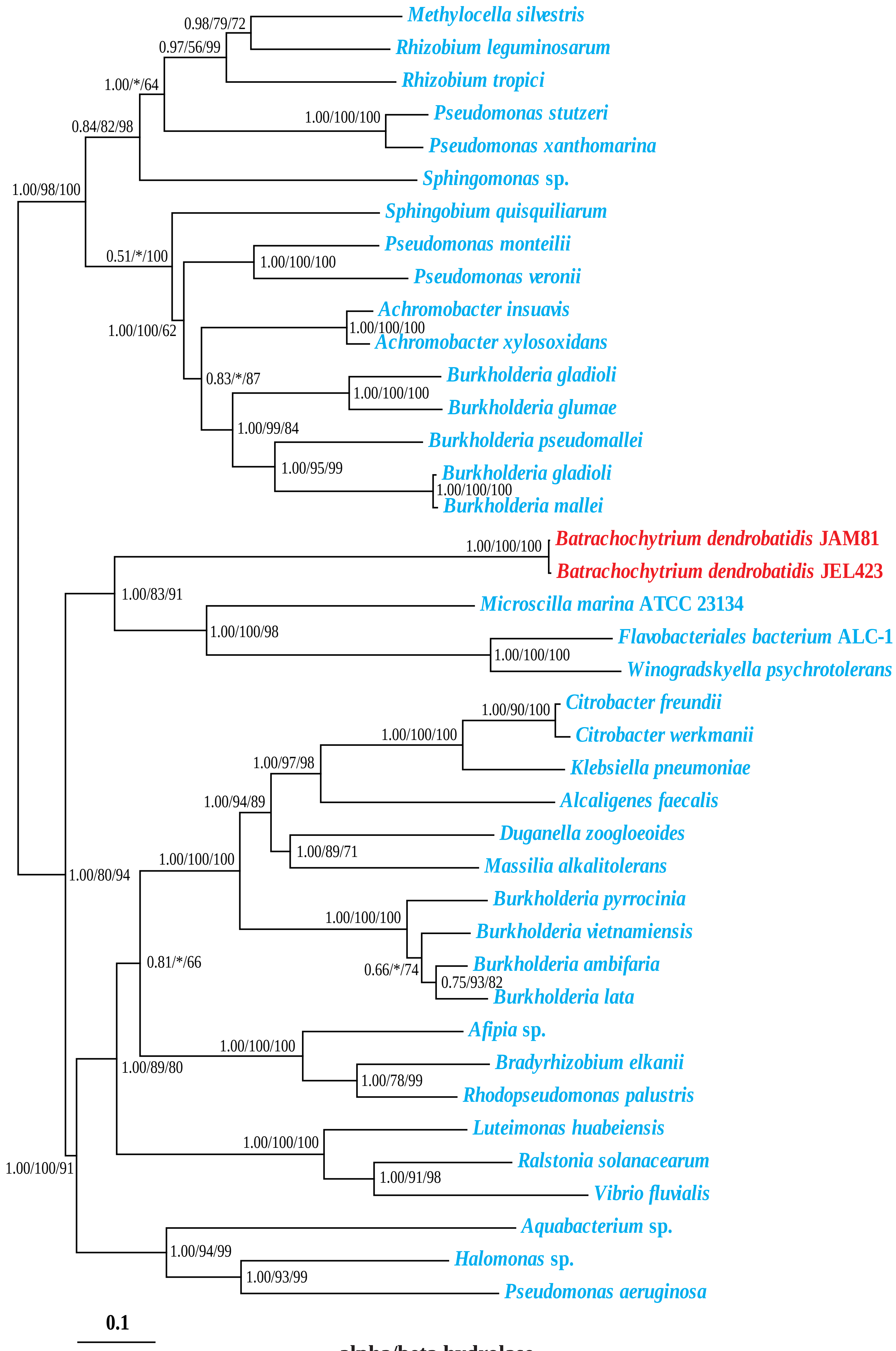

alpha/beta hydrolase

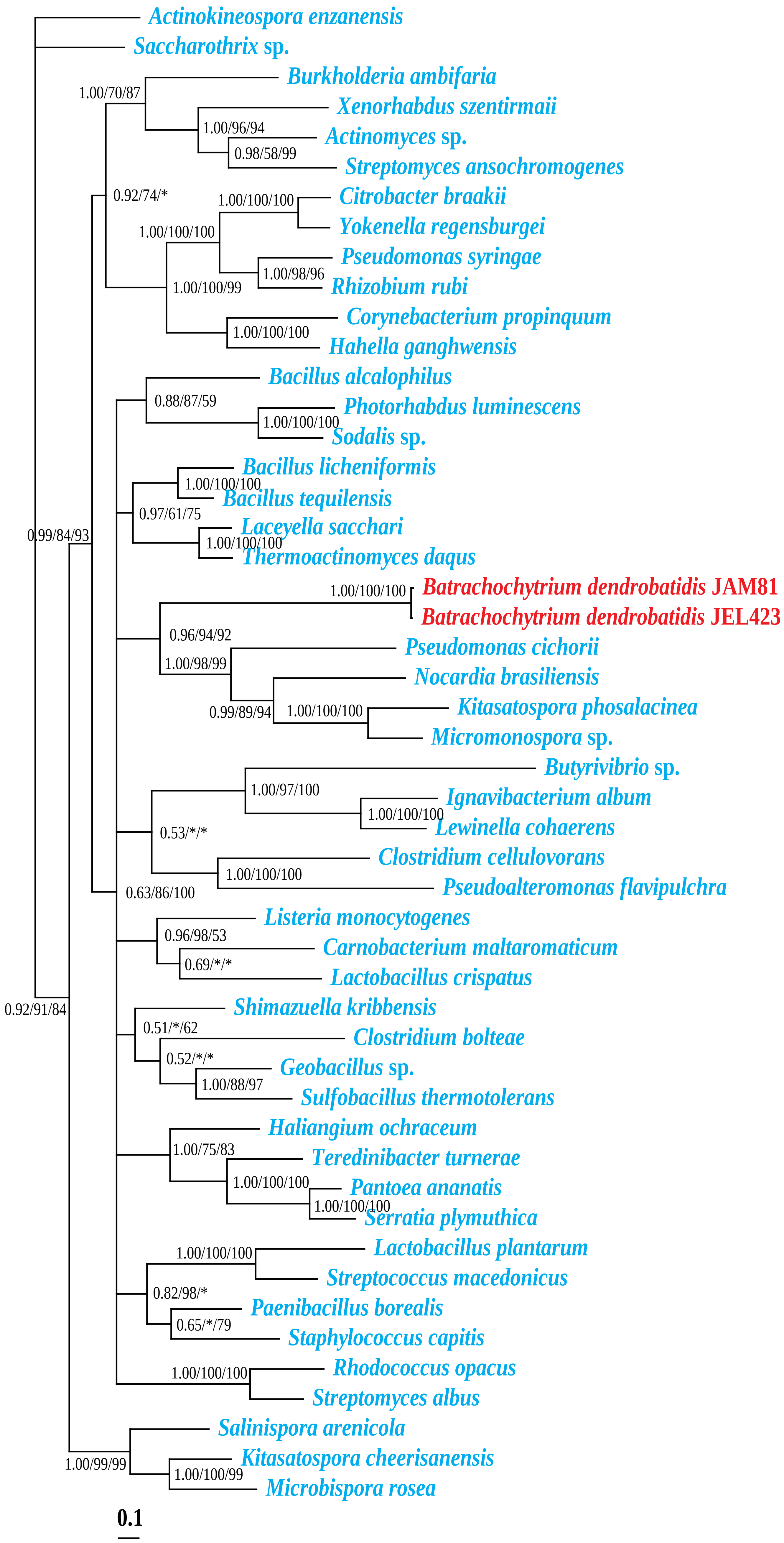

carboxylate-amine ligase

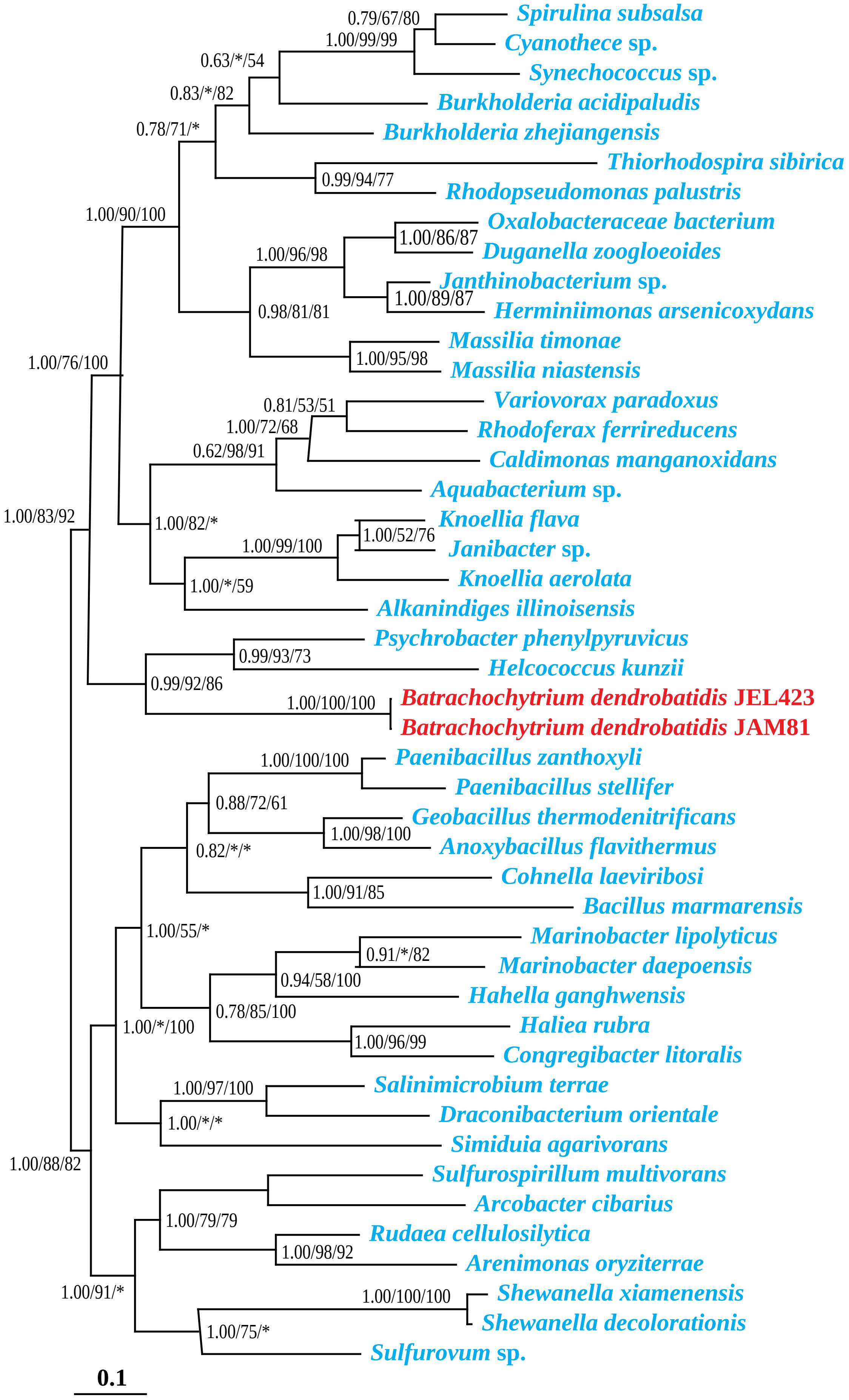

glutathione peroxidase

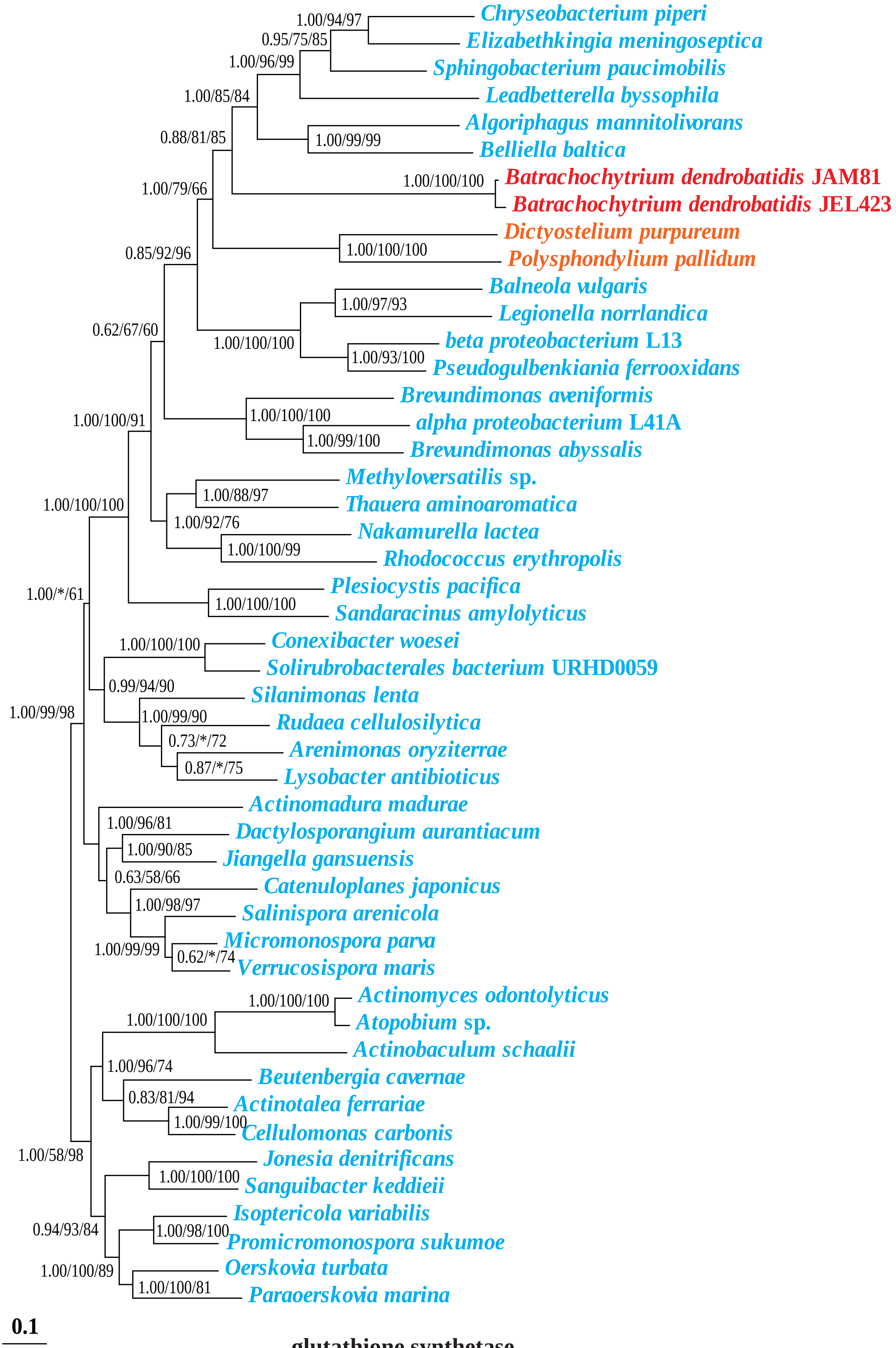

glutathione synthetase

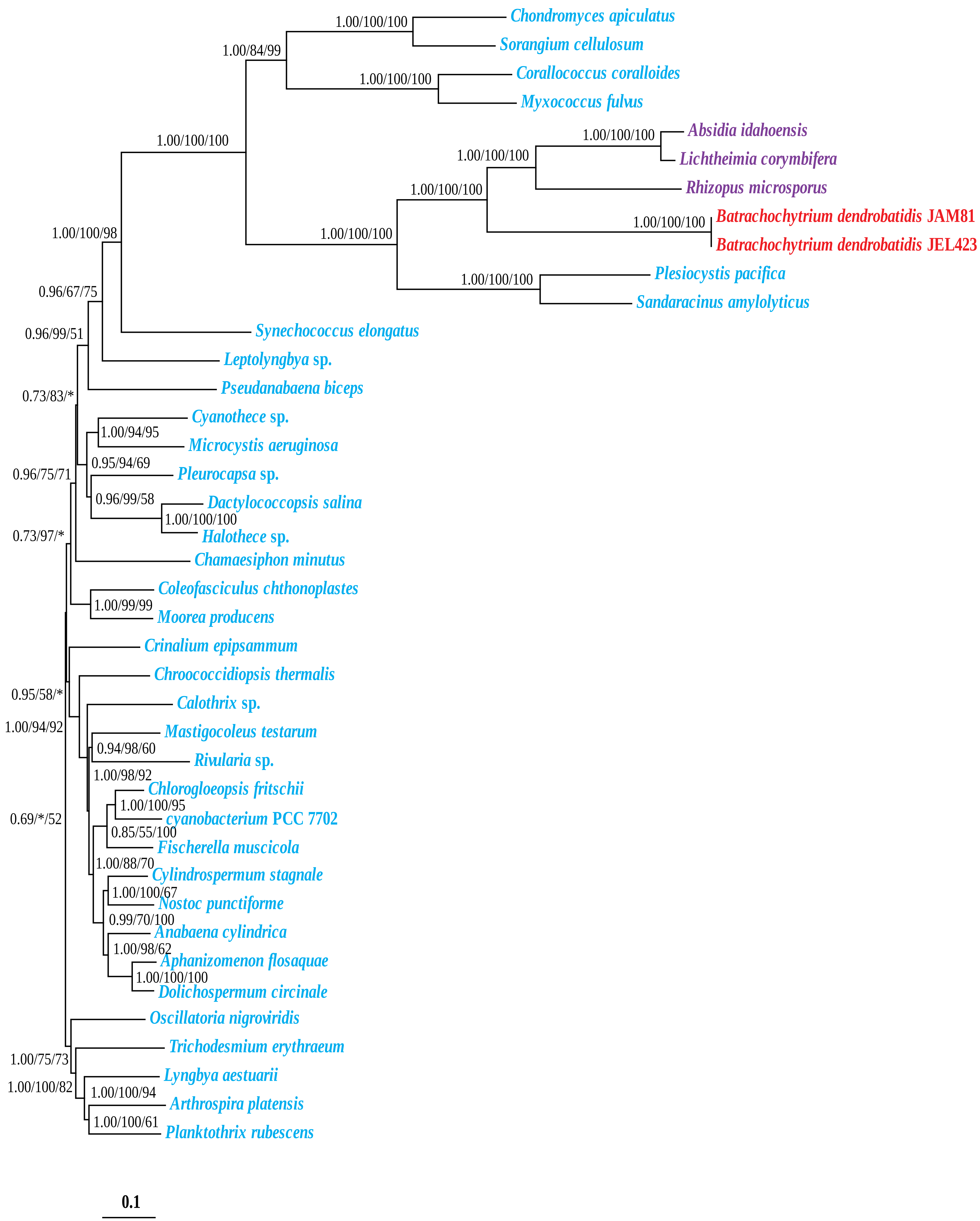

aminopeptidase

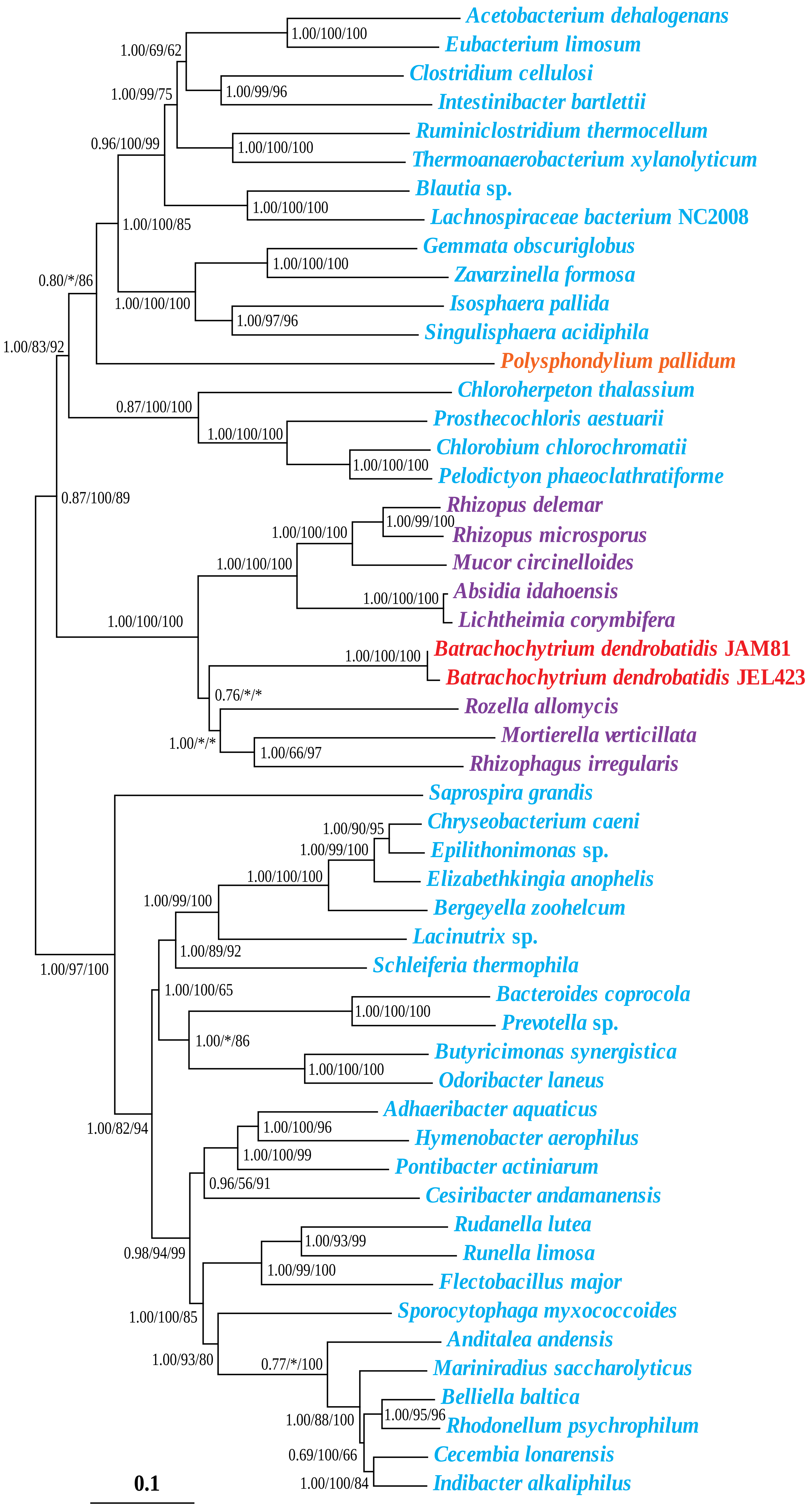

glutamine synthetase

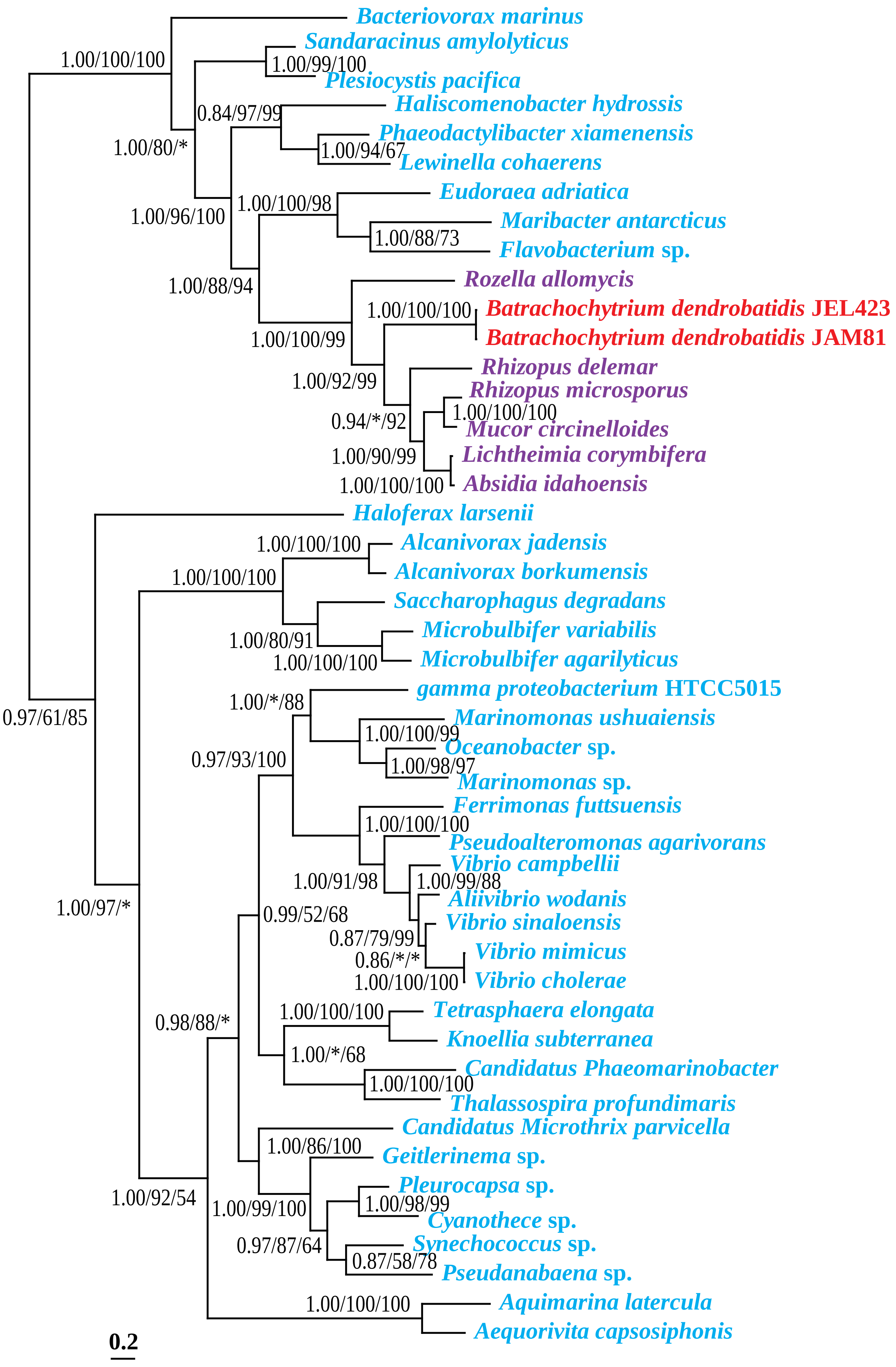

succinylglutamate desuccinylase

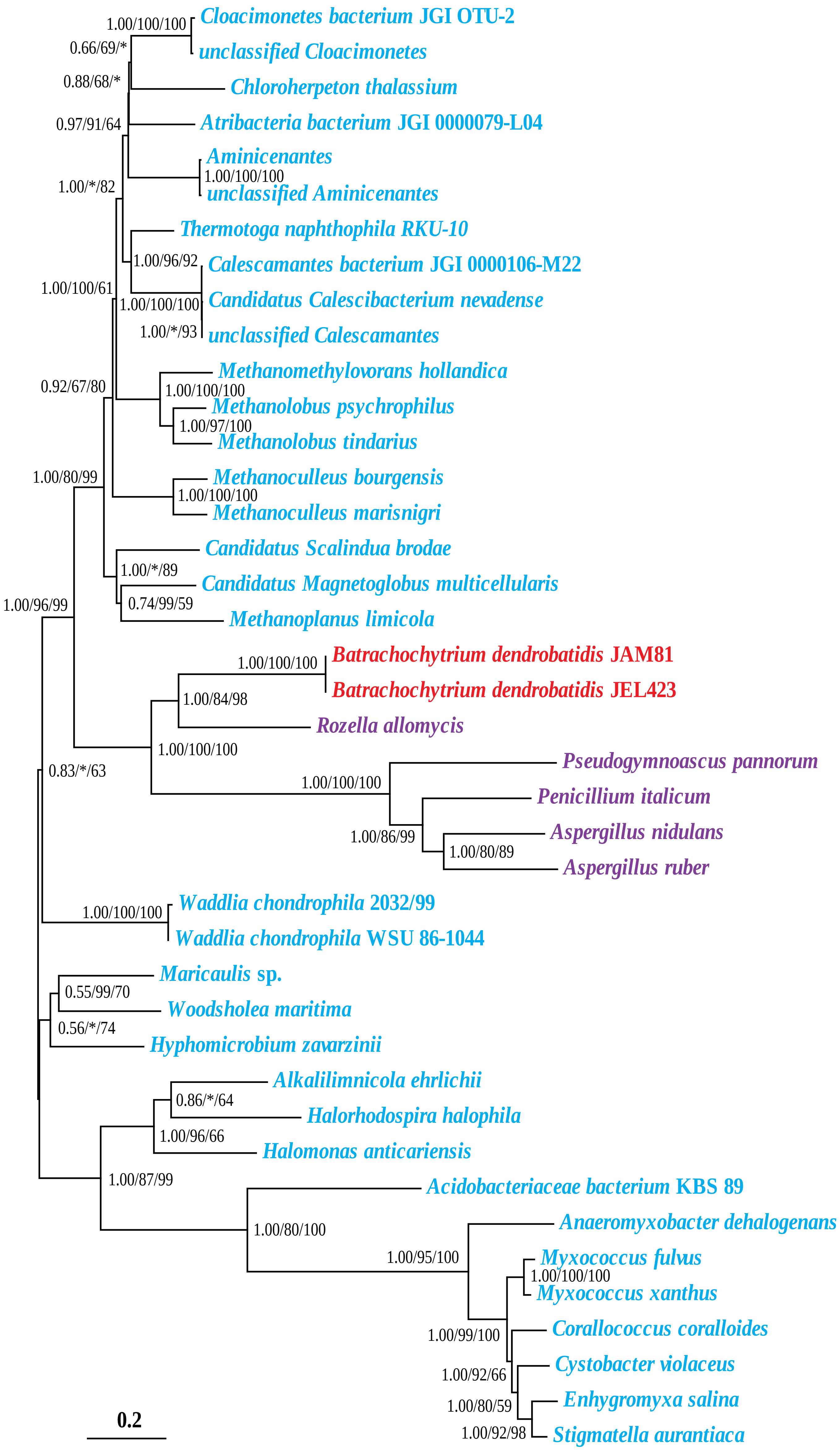

D-alanine--D-alanine ligase

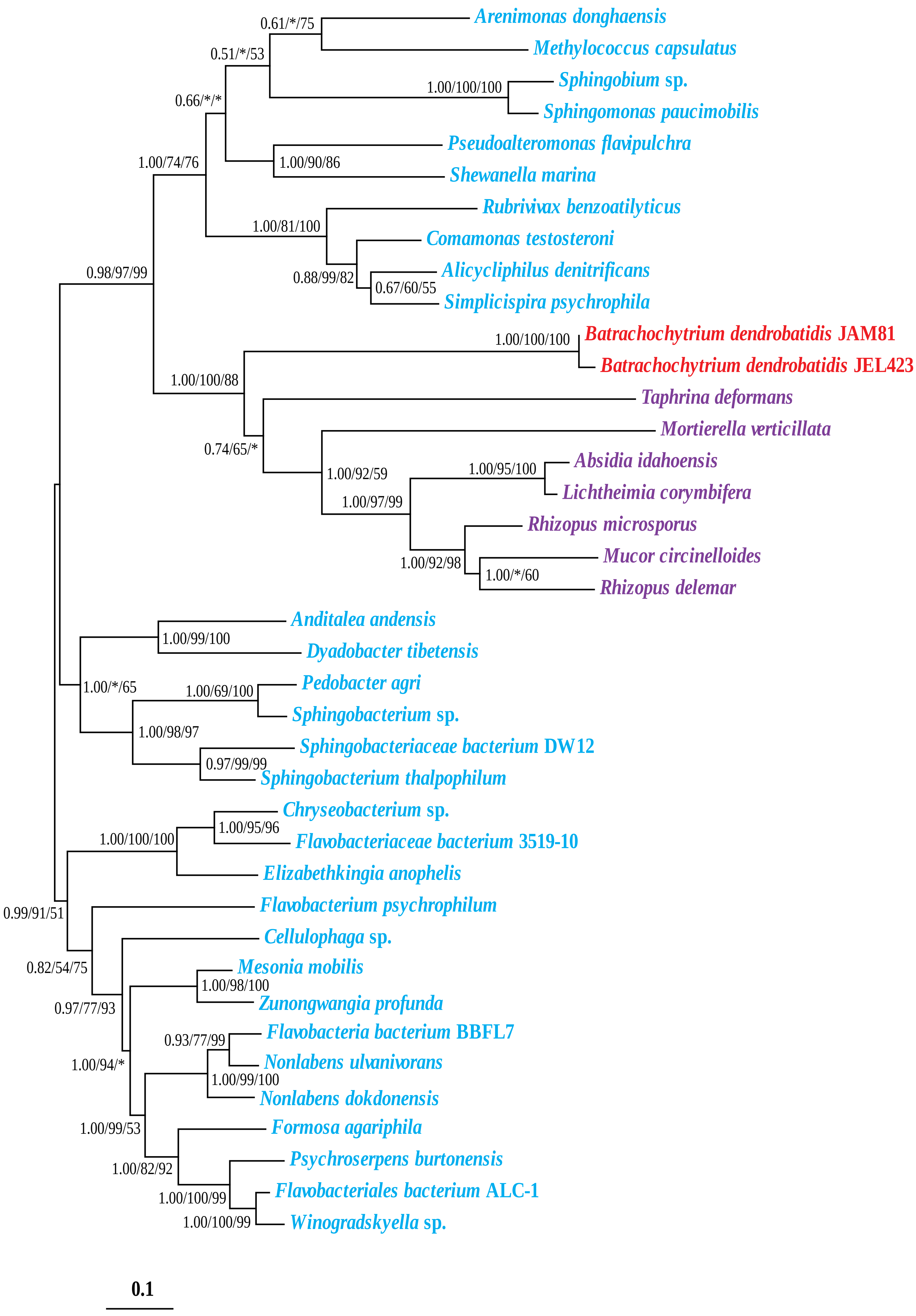

glutamyl-tRNA amidotransferase

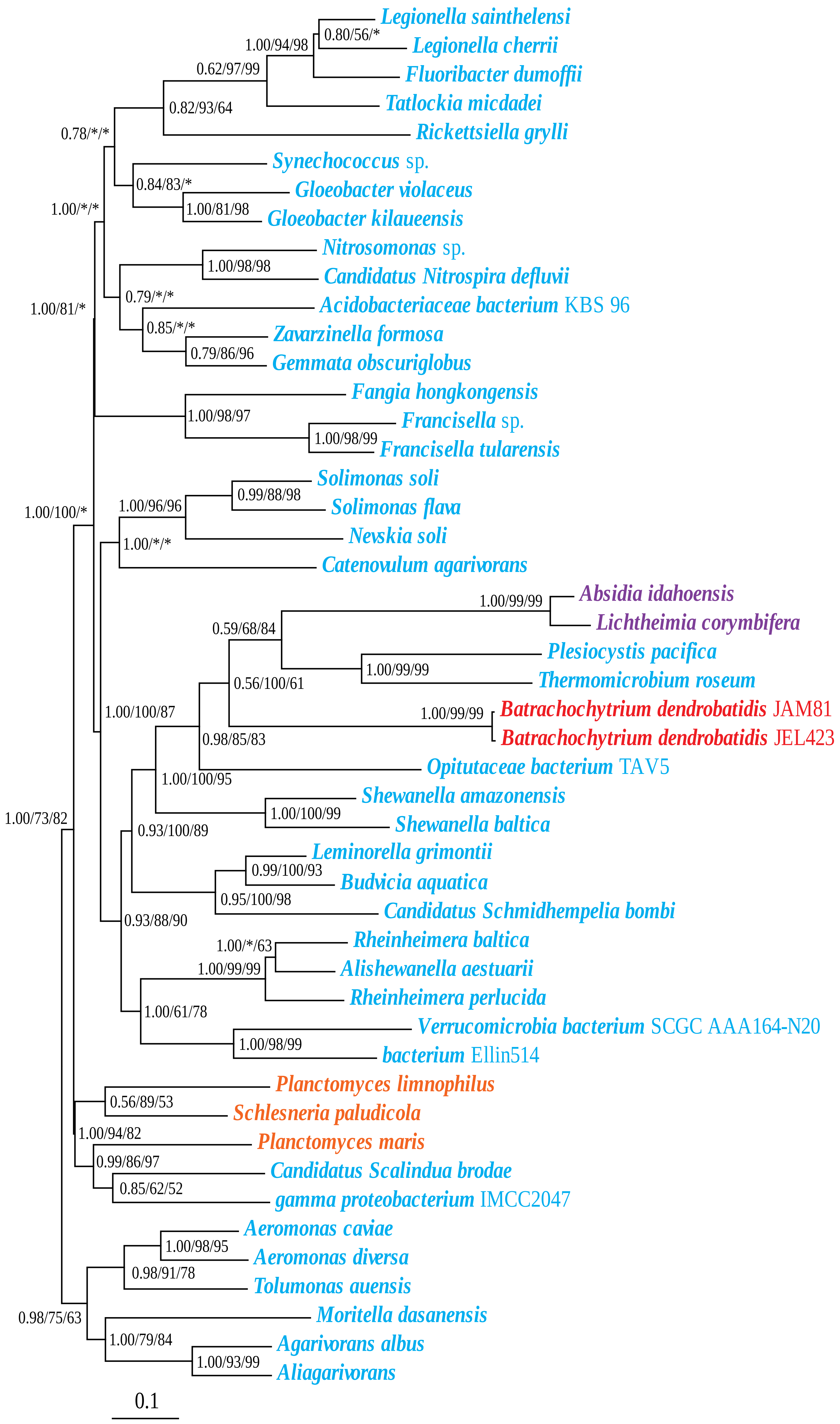

rhodanese-related sulfurtransferase

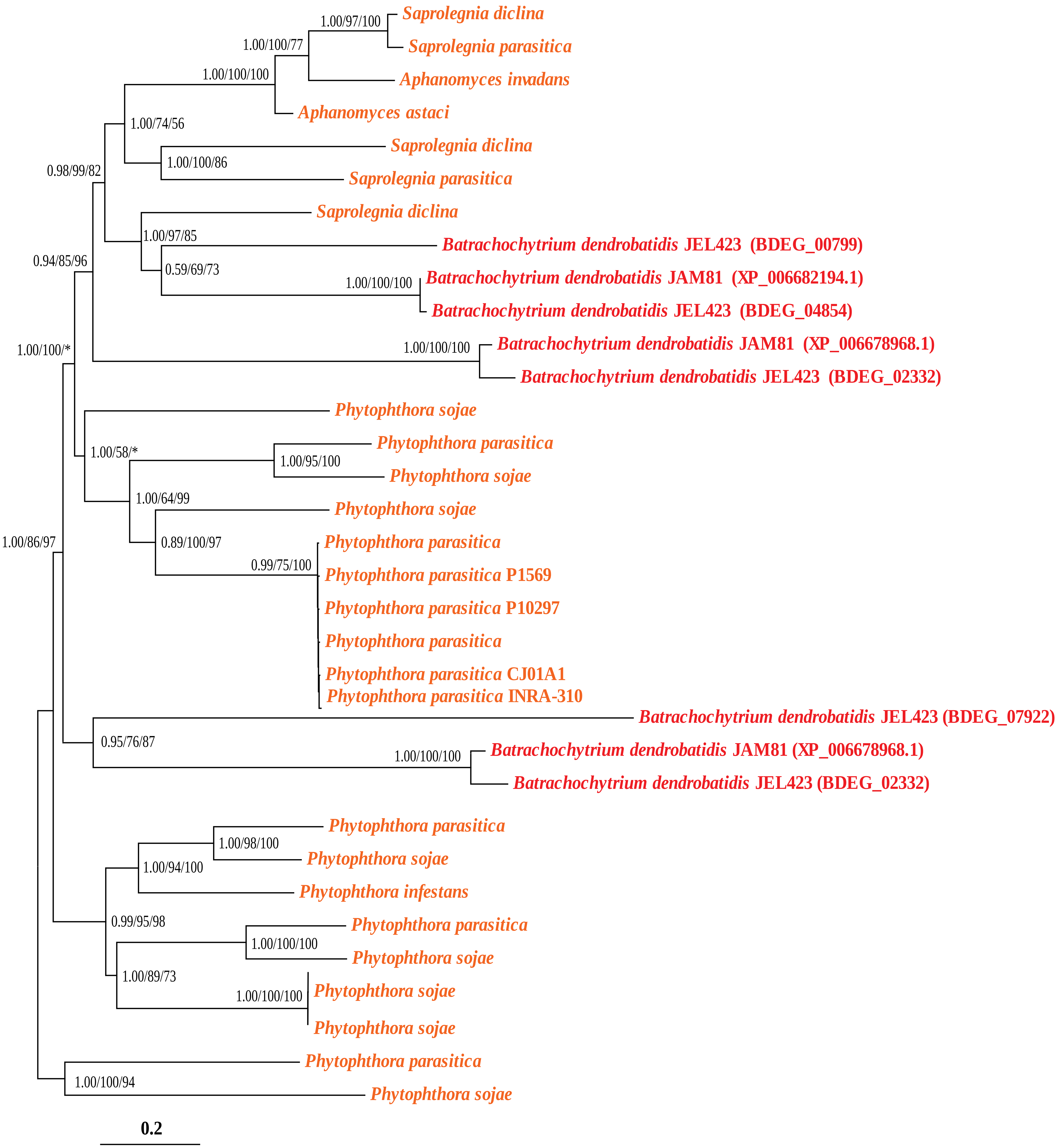

ankyrin repeat-containing protein
