# Supplementary material for: Contribution of Multiple Inter-Kingdom Horizontal Gene Transfers to Evolution and Adaptation of Amphibian-Killing Chytrid, Batrachochytrium dendrobatidis
Source: Front Microbiol. 2016 Aug 31;7:1360. doi: 10.3389/fmicb.2016.01360 (PMC5005798; doi:10.3389/fmicb.2016.01360)
Supplement: Table S1 — The code (script) and parameters used in the bioinformatic pipeline. [file Table1.docx]

Table S1. The code (script) and parameters used in the bioinformatic pipeline

| Step | Specific workflow | code (script) or parameters |
| --- | --- | --- |
| 1 | blast against fungi database | ** format fungi protein database for BLAST  formatdb -i fungi.fasta -p T -o T -n fungi  ** blast of *Bd* protein sequences against fungi  blastall -p blastp -d fungi -i Bd.fasta -e 1e-10 -b 1000 -a 4 -m8 -o Bd.out |
| 2 | screen genes present in five or fewer fungi | ** get the map of fungi Refseq ID to species  cat fungi.fasta\|grep "^>"\|sed -e 's/^>//'\|sed -e 's/\|.*\[/\[/'>fungi-id2species  ** extracting the ID with homology sequences in five or fewer fungi  awk -v OFS="\t" 'NR==FNR{x[$1]=$0}NR>FNR{print $0,x[$2]}' fungi-id2species Bd.out\|cut -f1,14\|awk '!a[$0]++'\|awk -v OFS="\t" '{a[$1]++}END{for(i in a) print i ,a[i]}'\|awk '$2<=5'\|cut -f1 >candi-id  ** extracting the fasta sequence of candidate (with homology sequences in five or fewer fungi)  formatdb -i bd.fasta -p T -o T -n bd  fastacmd -d bd -i candi-id -o candi.fasta |
| 3 | blast against nonfungi database | ** format non-fungi protein database for BLAST  formatdb -i non-fungi.fasta -p T -o T -n non-fungi  ** blast of Bd protein sequences against non-fungi  blastall -p blastp -d non-fungi -i candi.fasta -e 1e-10 -b 1000 -a 4 -m8 -o candi.out |
| 4 | detecting candidates with homology sequences in more than 20 non-fungal species | ** get the map of non-fungi Refseq ID to species  cat non-fungi.fasta\|grep "^>"\|sed -e 's/^>//'\|sed -e 's/\|.*\[/\[/'>non-fungi-id2species  ** extracting the ID with homology sequences in non-fungi  awk -v OFS="\t" 'NR==FNR{x[$1]=$0}NR>FNR{print $0,x[$2]}' non-fungi-id2species\|cut -f1,14\|awk '!a[$0]++'\|awk -v OFS="\t" '{a[$1]++}END{for(i in a) print i ,a[i]}'\|awk '$2>=20'\|cut -f1 >trans-id  ** extracting the fasta sequence of candidate (with homology sequences in more than 20 non-fungal species)  fastacmd -d bd -i trans-id -o trans.fasta |
| 5 | getting the homology sequences of each possible transferred gene | ** to each candidate transferred sequence:  blastall -p blastp -d nr -i trans.fasta -e 1e-10 -b 1000 -a 4 -m8 -o trans.out  cat trans.out\|cut -f2\|fastacmd -d nr -i - -o trans-ID.fasta |
| 6 | multiple alignments | with the default parameters in ClustalW2 |
| 7 | constructing the phylogenetic trees | Bayesian inference tree: with MrBayes 3.1.2  Mcmcp ngen=10000000 samplefreq=100 printfreq=1000 nchains=4  startingtree=random savebrlens=yes.  other parameters including prset aamodelpr, statefreqpr, Lset rates were adjusted based on the Prottest result.  ML tree: with online Phyml 3.1 (http://www.atgc-montpellier.fr/phyml/), the Substitution model was adjusted based on the Prottest result.  The bootstrap number is 1000.  NJ tree: with MEGA6, number of bootstrap replications: 1000 |
